# A survey of prevalence of narrative and systematic reviews in five major medical journals

**DOI:** 10.1186/s12874-017-0453-y

**Published:** 2017-12-28

**Authors:** Clovis Mariano Faggion, Nikolaos P. Bakas, Jason Wasiak

**Affiliations:** 1 Department of Periodontology and Operative Dentistry, Faculty of Dentistry, University of Münster, Waldeyerstraße 30, 48149 Münster, Germany; 2 School of Architecture, Engineering, Land and Environmental Sciences, Neapolis University Pafos, Paphos, Cyprus; 3 Melbourne School of Health Sciences, Department of Nursing, Faculty of Medicine, Dentistry and Health Sciences, University of Melbourne, Melbourne, Australia

**Keywords:** Review, Systematic review, Bias, Methodological study, Journal impact factor

## Abstract

**Background:**

Systematic reviews may provide less biased evidence than narrative reviews because they observe a strict methodology, similarly to primary studies. Hence, for clinical research questions, systematic reviews should be the study design of choice. It would be important to evaluate the prevalence and characteristics of narrative and systematic reviews published in prominent medical journals. Researchers and clinicians give great value to articles published in such scientific journals. This study sought to evaluate the prevalence and characteristics of narrative and systematic reviews in the five highest-ranked general medical journals and investigate the associations among type of review, number of citations, and impact factor (IF).

**Methods:**

We surveyed the five highest-ranked medical journals (*The New England Journal of Medicine, The Lancet, The Journal of the American Medical Association, The BMJ, and Annals of Internal Medicine*) for narrative and systematic reviews published between June 2015 and June 2016. We independently selected and extracted the data from the reviews by strictly following the pre-determined eligibility criteria (Systematic and narrative reviews that focused on the management of diseases). We conducted regression analyses to investigate the associations among review type, number of citations, and IF. We also descriptively reported narrative reviews containing some methodology that might be reproducible.

**Results:**

Two hundred seventy-five reviews were included: 75 (27%) systematic; 126 (46%) narrative with some methodology reported, and 74 (27%) narrative reviews. In comparison to systematic reviews, narrative reviews were more frequently published in journals with higher IF (risk ratio [RR] = 1.114 (95% CI 1.080 to 1.149). Systematic reviews received more citations than narrative reviews (group formed by narrative and narrative with some methodology reported (RR = 0.985 95% CI 0.978 to 0.991).

**Conclusions:**

Non-systematic evidence is the most prevalent type of evidence in reviews published in the five highest-ranked general medical journals. Narrative reviews were more frequently published in journals with higher IF. We recommend that journals limit their space for narrative information, and to address clinical research questions, these journals consider publishing systematic evidence exclusively.

**Electronic supplementary material:**

The online version of this article (10.1186/s12874-017-0453-y) contains supplementary material, which is available to authorized users.

## Background

Systematic reviews are considered the best way to synthesize evidence. This kind of review is developed with robust methodology to ensure that the review is reproducible. In contrast, narrative reviews do not include any pre-defined methodology or structure, which renders the reproduction of different steps challenging or impossible. One can argue that a narrative review may be inherently biased and therefore should not be viewed as a scientific work. What is the usefulness of research that cannot be reproduced? This rationale may be in line with recent efforts to prevent the wasteful use of research-related resources [1].

Evidence suggests that narrative reviews are frequently cited in specific medical disciplines such as dentistry [2–4]. This study sought to evaluate the proportion and characteristics of narrative and systematic reviews in major medical journals. These journals are well regarded by both researchers and clinicians who use information from these journals for their research and clinical decision-making. The aim of this study was to elucidate the current practice of these journals in publishing systematic and non-systematic evidence in the form of reviews.

Therefore, we scrutinized the five highest-ranked general medical journals (as classified by impact factor (IF) since 2015 in the annual Journal Citation Report (JCR) published by Thomson Reuters) for narrative or systematic reviews. We also evaluated the association between number of citations of the selected articles or journal IF and type of review.

## Methods

### Eligibility criteria

Systematic and narrative reviews that focused on the management of diseases and had been published in one of five major medical journals were selected for analysis. These journals were chosen because we believe they publish a higher proportion of studies/reviews with scientific merit and clinical relevance. Reviews on other topics such as methodological issues and statistics were excluded. Analysis and commentary documents were also excluded. The medical journals searched were: *The New England Journal of Medicine* (*NEJM*; IF:59.558), *The Lancet (Lancet)* (IF:44.002), *The Journal of the American Medical Association* (*JAMA*) (IF:37.684), *The British Medical Journal* (*BMJ*) (IF:19.697), and *Annals of Internal Medicine* (*AIM*) (IF:16.593). We also categorized reviews with a population health or policy-making (PHPM) perspective.

### Types of reviews: Definitions

Reviews in this study were classified as one of three types:

*Systematic review*: We considered a review “systematic” when authors explicitly reported the intention to perform a systematic review in the title, abstract or any part of the text.

*Narrative review with some methodology reported (narrative review-SMR)*: Authors report some steps that might be reproducible (e.g., data search and selection). The inclusion of search strategy and selection criteria is a requirement for publishing reviews in some journals [5, 6].

*Narrative review*: No report of the methodology used to perform the review (e.g., procedures for data search and selection). No part of the review can be reproduced.

### Search strategy

We obtained the International Standard Serial Numbers (ISSN) of the journals from the National Library of Medicine (NLM). The ISSN facilitates a focused and precise search. The ISSN numbers were: 0028–4793 for *NEJM*, 0140–6736 for *Lancet*, 0098–7484 for *JAMA*, 0959–8138 for *BMJ*, and 0003–4819 for *AIM*. A structured literature search was performed in MEDLINE via PubMed using a combination of ISSN numbers and the following keywords: *therapeutics OR therapeutic OR therapy OR therapies OR treatment OR treatments*. Finally, we applied the following PubMed filters: “review”, “systematic reviews”, and “custom date range (01 June 2015 to 01 June 2016)”.

### Data selection and extraction

Two authors (CMF, JW) independently selected the reviews by strictly following the pre-determined eligibility criteria. Two authors (CMF, NB) independently extracted information using a data extraction sheet developed by the first author that related to the following variables: 1) number of citations in Google Scholar received by different types of reviews (citations retrieved in the period between 23 and 27 August 2017); 2) number and proportion of different types of reviews published in the five medical journals included in this sample. We organized the reviews in two types (regarding their purpose): 1) clinically oriented (CO): reviews related to a clinical research question; 2) population health or policy making oriented (PHPM): reviews with a population perspective or more focused on the discussion of relevant topics. We used the following criteria to organize the reporting of methodology in the reviews: search strategy, data selection, data extraction, and quality/risk of bias (ROB) of primary studies included in the reviews. Disagreements on data selection and extraction procedures were resolved by discussion until consensus was achieved.

### Data analysis

We reported the distribution of reviews across the five medical journals and characteristics regarding type of review (narrative, systematic, or narrative review-SMR), the number of citations received (median and range), and IF (median and range). We checked types of reviews published in the five medical journals (which have different IF) and calculated the average IF (from 2015). After fitting a multinomial logistic regression model, we calculated risk ratios (RR) for dichotomous outcomes with respective 95% confidence intervals (CI). For this calculation, we grouped narrative reviews and narrative reviews-SMR into one group (narrative*). Furthermore, the associations were investigated inversely, using linear regression with type of review (narrative* = 1, systematic = 0) as predictor, and the number of citations and journal impact factor as dependent variables. Given that the predictor is dichotomous, the chi^2^ test was utilized (instead of the *p*-value) as significance measure. All data analysis was performed with MATLAB version R2016b (MathWorks Inc., Natick, Massachusetts).

## Results

Our initial search yielded 372,245 documents. After use of the PubMed filters, 399 of these were selected. After independent scrutiny of the titles and abstracts, we excluded an additional 124 documents because they failed to meet the methodological definition of a systematic, narrative review-SMR, or narrative review (list of excluded articles with reasons for exclusion in the Additional file 1). A total of 275 reviews were ultimately included. The full list of articles included in this study is reported in the Additional file 2.

**Fig. 1:**
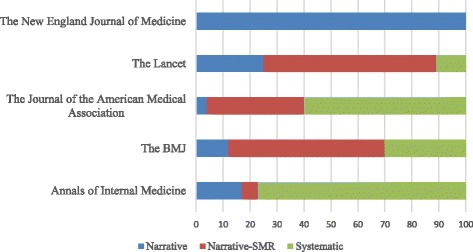
Distribution of types of reviews in the five medical journals (clinically oriented and population health/policy-making reviews presented together)

**Fig. 2:**
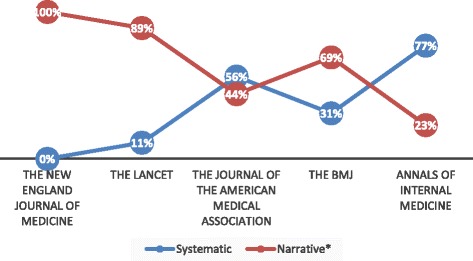
Percentage of reviews (systematic and narrative* (narrative reviews plus narrative reviews-SMR) in the five medical journals)

Table 1 and Fig. 1 report the distribution of reviews across the five medical journals examined. Most reviews were CO narrative reviews-SMR (*N* = 117, 43%), followed by CO systematic reviews (*N* = 73, 26%), CO narrative reviews (*N* = 51, 19%), PHPM narrative reviews (*N* = 23, 8%), PHPM narrative reviews-SMR (*N* = 9, 3%), and PHPM systematic reviews (N = 2, 1%). *Lancet* contributed the most reviews (*N* = 118, 43%). *JAMA* contributed the fewest reviews (*N* = 25, 9%). *Lancet* published the most CO narrative reviews-SMR (*N* = 70, 25%), and *NEJM* published the most CO narrative reviews (*N* = 30, 11%). *NEJM* contributed only narrative reviews to the present sample. Figure 2 reports the percentage of the different types of reviews (narrative* and systematic) published in each of the five journals. Narrative* reviews include narrative reviews plus narrative reviews-SMR. Table 2 presents the characteristics of narrative* and systematic reviews. Following the pre-defined evaluation criteria, 126 narrative reviews (63%) of this sample reported information on how authors performed the search of literature.

**Table 1:** Distribution of reviews across the five medical journals. Numbers in parentheses are percentages

Journal	Types of review					
	CO-Narrative	CO-Narrative with some method.	CO-Systematic	PHPM-Narrative	PHPM-Narrative with some method.	PHPM-Systematic
The New England Journal of Medicine	30 (100)	0	0	0	0	0
The Lancet	6 (5)	70 (59)	12 (10)	23 (20)	6 (5)	1 (1)
The Journal of the American Medical Association	1 (4)	9 (36)	14 (56)	0	1 (4)	0
The BMJ	8 (12)	36 (54)	19 (29)	0	2 (3)	1 (2)
Annals of Internal Medicine	6 (17)	2 (6)	28 (77)	0	0	0

*CO* clinically oriented, *PHPM* population health/policy-making

Narrative with some method. Means that the narrative reviews in this sample met one of the criterion (search strategy) that is potentially reproducible

**Table 2:** Characteristics of reviews included in this study. Numbers in parenthesis are percentages

Characteristics	Systematic review	Narrative review
Clinically oriented (CO) issue^a^	73 (97)	168 (84)
PHPM^b^ issue	2 (3)	32 (16)
Number of citations	5461	10,000
Methodology reported?		
• Search	4 (5)	126 (63)
• Selection	0 (0)	0 (0)
• Extraction	0 (0)	0 (0)
• Quality assessment	0 (0)	0 (0)
• Search + selection	0 (0)	0 (0)
• Search + quality assessment	1 (1.5)	0 (0)
• Search + selection + extraction	3 (4)	0 (0)
• Search + selection + quality assessment	4 (5)	0 (0)
• Search + extraction + quality assessment	1 (1.5)	0 (0)
• Search + selection + extraction + quality assessment	60 (80)	0 (0)
• No methodology reported	2 (3)	74 (37)

^a^CO issue: any review intended to address the management of a clinical condition (including also any measure to prevent disease). It includes reviews analysing the effectiveness, side-effects of interventions or both, effectiveness of screening etc

^b^PHPM issue: any review intended to generate hypotheses or facilitate discussion in the medical community

The median number of citations was 45 (range = 2 to 311), 29.5 (range = 0 to 215), and 43 (range 9 to 388) for narrative, narrative reviews-SMR, and systematic reviews, respectively. The median number of citations for narrative* reviews was 35 (range 0 to 311). The median IF was 44 (range 17 to 60), 44 (range 17 to 44), and 20 (range 17 to 44) for narrative, narrative reviews-SMR, and systematic reviews, respectively.

Logistic regression analysis generated a RR for the citations of 0.985 (95% CI 0.978 to 0.991) and for the IF 1.114 (95% CI 1.080 to 1.149). The coefficient for the citations regression was −18.17 (*p* = 0.0308) and for IF 13.19 (*p* < 0.001).

## Discussion

The present findings suggest that highly ranked medical journals publish more narrative reviews than systematic reviews. In this sample of reviews, the majority of reviews addressing clinical questions are narrative or narrative reviews-SMR. Compared with systematic reviews, narrative and narrative reviews-SMR are more likely to be published in highly ranked medical journals with higher IF. Systematic reviews, however, receive more citations than narrative and narrative reviews-SMR.

In one study [7] that evaluated the publication of systematic reviews in orthopaedic journals, the authors hypothesized that the number of citations for systematic reviews published in leading orthopaedic journals would be greater than the number of citations for narrative reviews published in the same journal. The authors found that “rigorous” (in their evaluation) systematic reviews received more citations than non-rigorous systematic and narrative reviews. Another study [8] evaluated five journals that published the most systematic reviews among a sample of 170 clinical journals in various medical fields. The authors found that systematic reviews were cited more often than narrative reviews. Similar results were found in a meta-analysis [9] that determined which among a large sample of articles received the greatest number of citations (*N* = 2646). In that study, narrative reviews received fewer citations than other types of reviews. Our findings are in agreement with publications above, although some other studies report the opposite. For example, in the field of dentistry some studies revealed more numerous citations for narrative reviews in comparison to systematic reviews [2–4]. These findings are supported by another study published in the field of dermatology [10] that found that authors of clinical trials and narrative reviews do not frequently cite systematic reviews. Some methodological [11] and discipline related issues might explain this variability on the association type of review and number of citations.

The present results demonstrate that much information reported in reviews published in these five journals derives from potentially biased evidence. One might argue that all types of information are subject to bias, including studies with well-recognized methodology, such as systematic reviews. However, because these studies have a defined structure, they are vulnerable to audit and quality evaluation. For example, tools such as A Measurement Tool to Assess Systematic Reviews (AMSTAR) [12] and a tool to assess risk of bias in systematic reviews (ROBIS) [13] can be used to evaluate the methodological quality and risk of bias of systematic review domains. In contrast, narrative reviews do not allow for this type of evaluation because they do not have a structured framework. Therefore, evaluating the methodology and quality of narrative reviews is challenging, if not impossible. Our results are in fact an optimistic view of reality, taking into account that other types of articles (commentaries, opinion paper etc. which are not systematic) were not included in our analysis. Thus, we might have an even worst scenario on the evidence currently used to support clinical decisions.

The present study included a group of narrative reviews-SMR in order to differentiate studies with partially reproducible methodology from those without any reproducible methodology, i.e. narrative reviews. We understand this is the fairest judgement of articles that, at least, explicitly reported part of the methodology. Based on our evaluation criteria, these narrative reviews-SMR described the search strategy used in their reviews. In fact, most articles in this group come from *Lancet* and *BMJ* journals, where sections in the paper are entitled “search strategy and selection criteria” (*Lancet*) and “sources and selection criteria” (*BMJ*). The selection criteria section in these articles, however, does not seem to be reported in enough detail for repeatability and traceability [14]. Nevertheless, these reviews do not allow for the complete reproduction of several steps and may therefore be considered as narrative reviews based on non-systematic evidence.

In our study, narrative* reviews were more frequently published in journals with higher IF, as demonstrated by the regression coefficients. Furthermore, the reported RR indicates that the probability of a review being narrative* (compared to the probability of being systematic) increases 1.114 times for each unit increase in the journal IF. This outcome likely reflects the fact that all publications in the journal with the highest IF (*NEJM*) were narrative reviews. These findings are in agreement with outcomes in other medical fields, such as dentistry and oncology, in which journals with the highest IF [15, 16] tend to publish invited narrative reviews. Based on this information, we postulate that readers may not be aware of the importance of the methodology of reviews in such highly ranked journals. For readers, publication in a highly ranked journal might be sufficient to confirm the quality of the article. It is, however, important to emphasize that narrative reviews published in such high-level medical journals are authored by specialists in their fields. Thus, it is likely that the work will be high-quality. The articles in these journals must pass a strict and rigid peer-review process [17], a process that supports the delivery of reliable work. Furthermore, publishing in one of these journals is quite difficult. These journals have low acceptance rates, and some evidence suggests that low rates of manuscript acceptance appear to predict higher methodological quality scores [18]. Thus, one can consider high IF as a proxy for quality. Nevertheless, these are arguments based on the backgrounds of people and journals involved in the process, rather than facts that can be measured, verified and replicated [19]. A study’s quality should be determined on its own merits rather than on the background and quality of its authors.

The present article does not recommend the total elimination of narrative reviews. These types of reviews have a place in science, but they should not be the first option for scientists seeking information to address clinical questions. Narrative reviews could be used to facilitate discussion of relevant topics. Because of its lack of structure (and consequent invulnerability to audit), a narrative review should not be used as the basis for any strategy (therapeutic or otherwise) to address human disease. High-quality medical journals could implement a policy of limiting the space devoted to “non-systematic” literature. Information serving as the basis for further research or decision-making should be published only when a systematic and reproducible approach was used. This approach would help to prevent research based on biased information. Because the focus of our study was on reviews on interventions, we also organised the reviews on CO and PHPM. The idea was to provide the reader with a better picture when the focus of the review is to facilitate discussion, (PHPM) or to address management of diseases (CO). We understand that a review focusing on the management of diseases require a more systematic approach to avoid or to reduce the chances of biased results.

Some limitations of the present study should be discussed. Firstly, the study included a select group of medical journals. One can consider this information being representative to these five journals only. However, the literature search in only these five medical journals was the main inclusion criterion in our study, and it might be considered a “strength” due to the potential impact of the present findings on the research and medical community. As commented above, these journals are considered the best medical journals available, as indicated by their very high IF. Researchers will very likely use studies reported in these journals to plan and perform their own research. Similarly, clinicians will use the evidence reported in these journals to support their decisions on therapeutic approaches. Secondly, we conducted the literature search over a relatively short period of one year. Hence, we cannot extrapolate the findings to older reviews. However, there is no reason to believe that the pattern of publication of narrative, narrative reviews-SMR and systematic reviews has changed over time. Because the systematic review was developed more recently than the narrative review, one would expect these journals to have published a greater proportion of narrative reviews in earlier years. Furthermore, with the rise of evidence-based medicine in recent years, researchers and clinicians should be more aware of the importance of methodology. Such an awareness might result in a greater proportion of systematic reviews.

The present study also intends to encourage discussion on a global definition for systematic reviews. We used a definition based on the intention of authors to perform a systematic review, instead of definitions published in the literature [14, 20, 21] (see Additional file 3, systematic review definitions) which are all based on the perspectives of authors evaluating systematic reviews.

The main problem in defining systematic reviews is to determine thresholds for what is systematic and what is not. For example, two definitions [14, 20] report “reproducibility” as key characteristics of a systematic review. If this criterion is applied, many systematic reviews included in overviews would probably not be considered systematic. A recent publication showed that the information necessary to reproduce meta-analytic estimates was reported in only 65% of systematic reviews of biomedical interventions [22]. Many overviews of systematic reviews demonstrated that there is poor reporting of systematic reviews in different medical disciplines when the PRISMA checklist is applied [23–26]. Thus, this poor reporting would limit (or even make impossible) the reproducibility of the methodology, and this criterion would not be met for selecting the review as systematic. Furthermore, we understand that judging the review from the intention of authors to perform a systematic review leads to a more fair evaluation. One can argue that authors not explicitly reporting a systematic review would have an unfair judgement when quality tools are applied. For example, if authors of the review intend to publish a narrative review, but authors of the overview considered it as a systematic review. Hence, a narrative review would be judged as systematic. Finally, by including reviews with poor or no methodology, but named as systematic by review authors, clinicians and non-methodologist readers will have more comprehensive information to differentiate the robust from the methodologically poor systematic reviews. This information would then be provided in the quality/ROB evaluation of reviews with available methodological tools [12, 13].

A previous study [8] also compared systematic and narrative reviews in medical literature. They defined a review as systematic when “the authors had to clearly state the clinical topic of the review, how the evidence was retrieved and from what sources (i.e., naming the databases), and provide explicit inclusion and exclusion criteria.” Thus, these criteria would meet the criteria for defining a narrative review-SMR in our sample. One may consider that some reviews regarded as systematic by Montori et al. [8] could be, in fact, narrative reviews. We provided an analysis of narrative and systematic reviews (Table 2) to report to what extent systematic reviews are in fact systematic, by applying some criteria reported in published systematic review definitions.

## Conclusions

The present study demonstrated that narrative reviews are more prevalent than systematic reviews in highly ranked medical journals. Much of the evidence provided by these journals does not represent reproducible findings. We recommend that, to address clinical research questions, these journals consider publishing systematic evidence exclusively. Editors of medical journals should limit the amount of non-systematic evidence to reduce the probability of delivering biased information to readers. Further discussion on a consensus definition for systematic reviews is needed.

## Additional files

Additional file 1: Excluded articles. List of articles excluded with reasons for exclusion. (DOCX 54 kb)

Additional file 2: Included articles. List of articles included in this study. (DOCX 61 kb)

Additional file 3: Systematic review definitions. Definitions of systematic reviews published in the literature. (DOCX 14 kb)

## Abbreviations

AIM: Annals of Internal Medicine
AMSTAR: A Measurement Tool to Assess Systematic Reviews
BMJ: The British Medical Journal
CO: clinically oriented
IF: Impact factor
IQR: Interquartile range
ISSN: International Standard Serial Numbers
JAMA: The Journal of the American Medical Association
JCR: Journal citation report
MD: Mean difference
Narrative review-SMR: narrative review with some methodology reported
Narrative* review: it includes narrative reviews plus narrative reviews-SMR
NEJM: The New England Journal of Medicine
NLM: National Library of Medicine
NLM: National Library of Medicine
PHMP: population health or policy-making
ROBIS: tool to assess risk of bias in systematic reviews

## References

[1] Moher D, Glasziou P, Chalmers I, Nasser M, Bossuyt PM, Korevaar DA, Graham ID, Ravaud P, Boutron I (2016). Increasing value and reducing waste in biomedical research: who's listening?. Lancet.

[2] Fardi A, Kodonas K, Gogos C, Economides N (2011). Top-cited articles in endodontic journals. J Endod.

[3] Feijoo JF, Limeres J, Fernández-Varela M, Ramos I, Diz P (2014). The 100 most cited articles in dentistry. Clin Oral Investig.

[4] Faggion CM Jr, Málaga L, Monje A, Trescher AL, Listl S, Alarcón MA. The 300 most cited articles published in periodontology. Clin Oral Investig. 2016; [Epub ahead of print] 10.1007/s00784-016-1990-1 27844151

[5] Lancet Oncology. http://thelancet.com/lanonc/information-for-authors/article-types-manuscript-requirements. Accessed 26 January 2017.

[6] Lancet. http://www.thelancet.com/lancet/information-for-authors/article-types-manuscript-requirements. Accessed 26 January 2017.

[7] Bhandari M, Montori VM, Devereaux PJ, Wilczynski NL, Morgan D, Haynes RB (2004). Doubling the impact: publication of systematic review articles in orthopaedic journals. J Bone Joint Surg Am.

[8] Montori VM, Wilczynski NL, Morgan D, Haynes RB, Hedges Team (2003). Systematic reviews: a cross-sectional study of location and citation counts. BMC Med.

[9] Patsopoulos NA, Analatos AA, Ioannidis JP (2005). Relative citation impact of various study designs in the health sciences. JAMA.

[10] Conde-Taboada A, Aranegui B, García-Doval I, Dávila-Seijo P, González-Castro U (2014). The use of systematic reviews in clinical trials and narrative reviews in dermatology: is the best evidence being used?. Actas Dermosifiliogr.

[11] Ioannidis JP, Boyack K, Wouters PF (2016). Citation metrics: a primer on how (not) to normalize. PLoS Biol.

[12] Shea BJ, Grimshaw JM, Wells GA, Boers M, Andersson N, Hamel C, Porter AC, Tugwell P, Moher D, Bouter LM (2007). Development of AMSTAR: a measurement tool to assess the methodological quality of systematic reviews. BMC Med Res Methodol.

[13] Whiting P, Savović J, Higgins JP, Caldwell DM, Reeves BC, Shea B, Davies P, Kleijnen J, Churchill R, ROBIS group (2016). ROBIS: a new tool to assess risk of bias in systematic reviews was developed. J Clin Epidemiol.

[14] Haddaway NR, Land M, Macura B (2017). "a little learning is a dangerous thing": a call for better understanding of the term 'systematic review'. Environ Int.

[15] Periodontology 2000. http://onlinelibrary.wiley.com/journal/10.1111/(ISSN)1600-0757. Accessed 24 January 2017.

[16] CA: A Cancer Journal for Clinicians. http://onlinelibrary.wiley.com/journal/10.3322/(ISSN)1542-4863. Accessed 24 January 2017.

[17] Lee KP, Boyd EA, Holroyd-Leduc JM, Bacchetti P, Bero LA (2006). Predictors of publication: characteristics of submitted manuscripts associated with acceptance at major biomedical journals. Med J Aust.

[18] Lee KP, Schotland M, Bacchetti P, Bero LA (2002). Association of journal quality indicators with methodological quality of clinical research articles. JAMA.

[19] Jasny BR, Chin G, Chong L, Vignieri S (2011). Data replication & reproducibility. Again, and again, and again .... Introduction. Science.

[20] Page MJ, Shamseer L, Altman DG, Tetzlaff J, Sampson M, Tricco AC, Catalá-López F, Li L, Reid EK, Sarkis-Onofre R, Moher D (2016). Epidemiology and reporting characteristics of systematic reviews of biomedical research: a cross-sectional study. PLoS Med.

[21] Cochrane Glossary. http://community.cochrane.org/glossary. Accessed 04 September 2017.

[22] Page MJ, Altman DG, Shamseer L, McKenzie JE, Ahmadzai N, Wolfe D, Yazdi F, Catalá-López F, Tricco AC, Moher D. Reproducible research practices are underused in systematic reviews of biomedical interventions. J Clin Epidemiol. 2017; doi:10.1016/j.jclinepi.2017.10.017. [Epub ahead of print] 10.1016/j.jclinepi.2017.10.017 29113936

[23] Peters JP, Hooft L, Grolman W, Stegeman I (2015). Reporting quality of systematic reviews and meta-analyses of Otorhinolaryngologic articles based on the PRISMA statement. PLoS One.

[24] Gagnier JJ, Kellam PJ (2013). Reporting and methodological quality of systematic reviews in the orthopaedic literature. J Bone Joint Surg Am.

[25] Wasiak J, Tyack Z, Ware R, Goodwin N, Faggion CM Jr. Poor methodological quality and reporting standards of systematic reviews in burn care management. Int Wound J. 2017;14:754–63 10.1111/iwj.12692 PMC7949759 27990772

[26] Wasiak J, Shen AY, Ware R, O'Donohoe TJ, Faggion CM Jr. Methodological quality and reporting of systematic reviews in hand and wrist pathology. J Hand Surg Eur Vol. 2017;42:852–56 10.1177/1753193417712660 28610464

