# Supplementary material for: A survey of prevalence of narrative and systematic reviews in five major medical journals
Source: BMC Med Res Methodol. 2017 Dec 28;17:176. doi: 10.1186/s12874-017-0453-y (PMC5746017; doi:10.1186/s12874-017-0453-y)
Supplement: Supplementary file 1 — Excluded articles. List of articles excluded with reasons for exclusion. (DOCX 54 kb) [file 12874_2017_453_MOESM1_ESM.docx]

**Additional file 1**

**Excluded articles with reasons for exclusion**

1. Trinquart L, Attiche N, Bafeta A, Porcher R, Ravaud P. Uncertainty in Treatment Rankings: Reanalysis of Network Meta-analyses of Randomized Trials. Ann Intern Med. 2016;164:666-73. doi: 10.7326/M15-2521.

Reason: Overview of meta-analyses

2. Rouse B, Cipriani A, Shi Q, Coleman AL, Dickersin K, Li T. Network Meta-analysis for Clinical Practice Guidelines: A Case Study on First-Line Medical Therapies for Primary Open-Angle Glaucoma. Ann Intern Med. 2016;164:674-82. doi: 10.7326/M15-2367.

Reason: Guideline article

3. Winawer SJ(1), Fischer SE(1), Levin B(2). Evidence-Based, Reality-Driven Colorectal Cancer Screening Guidelines: The Critical Relationship of Adherence to Effectiveness. JAMA. 2016;315:2065-6. doi: 10.1001/jama.2016.3377.

Reason: Viewpoint article/methodology article

4. Deyo RA, Mirza SK. CLINICAL PRACTICE. Herniated Lumbar Intervertebral Disk. N Engl J Med. 2016;374:1763-72. doi: 10.1056/NEJMcp1512658.

Reason: case-report+review+guideline

5. Qaseem A, Kansagara D, Forciea MA, Cooke M, Denberg TD; Clinical Guidelines. Management of Chronic Insomnia Disorder in Adults: A Clinical Practice Guideline. Committee of the American College of Physicians. Ann Intern Med. 2016 May 3. doi: 10.7326/M15-2175. [Epub ahead of print]

Reason: Guideline

6. Phillips JP. Workplace Violence against Health Care Workers in the United States. N Engl J Med. 2016;374:1661-9. doi: 10.1056/NEJMra1501998.

Reason: Not prevention or intervention to address a disease

7. Duan-Porter W, Goldstein KM, McDuffie JR, Hughes JM, Clowse ME, Klap RS, Masilamani V, Allen LaPointe NM, Nagi A, Gierisch JM, Williams JW Jr. Reporting of Sex Effects by Systematic Reviews on Interventions for Depression, Diabetes, and Chronic Pain. Ann Intern Med. 2016 Apr 26. doi: 10.7326/M15-2877. [Epub ahead of print]

Reason: Overview of SRs

8. Goyal M, Menon BK, van Zwam WH, Dippel DW, Mitchell PJ, Demchuk AM, Dávalos A, Majoie CB, van der Lugt A, de Miquel MA, Donnan GA, Roos YB, Bonafe A, Jahan R, Diener HC, van den Berg LA, Levy EI, Berkhemer OA, Pereira VM, Rempel J, Millán M, Davis SM, Roy D, Thornton J, Román LS, Ribó M, Beumer D, Stouch B, Brown S, Campbell BC, van Oostenbrugge RJ, Saver JL, Hill MD, Jovin TG; HERMES collaborators..

Endovascular thrombectomy after large-vessel ischaemic stroke: a meta-analysis of individual patient data from five randomised trials. Lancet. 2016;387:1723-31. doi: 10.1016/S0140-6736(16)00163-X.

Reason: Individual patient data; Meta-analysis of only 5 trials

9. Chamberlain JJ, Rhinehart AS, Shaefer CF Jr, Neuman A. Diagnosis and Management of Diabetes: Synopsis of the 2016 American Diabetes

Association Standards of Medical Care in Diabetes. Ann Intern Med. 2016;164:542-52. doi: 10.7326/M15-3016.

Reason: Guideline

10. Dowell D, Haegerich TM, Chou R. CDC Guideline for Prescribing Opioids for Chronic Pain--United States, 2016. JAMA. 2016;315:1624-45. doi: 10.1001/jama.2016.1464.

Reason: Guideline + updated systematic review

11. Vinik AI. CLINICAL PRACTICE. Diabetic Sensory and Motor Neuropathy.

N Engl J Med. 2016;374:1455-64. doi: 10.1056/NEJMcp1503948.

Reason: Mixture of case-report + review + guideline

12. Chan JC, Gregg EW, Sargent J, Horton R. Reducing global diabetes burden by implementing solutions and identifying gaps: a Lancet Commission. Lancet. 2016;387:1494-5. doi: 10.1016/S0140-6736(16)30165-9.

Reason: Opinion/analysis paper

13. Smetana GW, Cutlip DE, Pinto DS. Should We Screen for Coronary Heart Disease in Asymptomatic Persons?: Grand Rounds Discussion From Beth Israel Deaconess Medical Center. Ann Intern Med. 2016;164:479-87. doi: 10.7326/M15-2910.

Reason: Discussion paper

14. Howard G, Roubin GS, Jansen O, Hendrikse J, Halliday A, Fraedrich G, Eckstein HH, Calvet D, Bulbulia R, Bonati LH, Becquemin JP, Algra A, Brown MM, Ringleb PA, Brott TG, Mas JL; Carotid Stenting Trialists' Collaboration.. Association between age and risk of stroke or death from carotid endarterectomy and carotid stenting: a meta-analysis of pooled patient data from four randomised trials. Lancet. 2016;387:1305-11. doi: 10.1016/S0140-6736(15)01309-4.

Reason: Meta-analysis with individual patient data with 4 trials only

15. Stone GW, Gao R, Kimura T, Kereiakes DJ, Ellis SG, Onuma Y, Cheong WF, Jones-McMeans J, Su X, Zhang Z, Serruys PW. 1-year outcomes with the Absorb bioresorbable scaffold in patients with coronary artery disease: a patient-level, pooled meta-analysis. Lancet. 2016;387:1277-89. doi: 10.1016/S0140-6736(15)01039-9.

Meta-analysis with individual patient data with 4 trials only

16. Ohman EM. CLINICAL PRACTICE. Chronic Stable Angina. N Engl J Med. 2016;374:1167-76. doi: 10.1056/NEJMcp1502240.

Reason: Mixture of case-report + review + guideline

17. Jain A, Cifu AS. Prevention, Diagnosis, and Treatment of Postthrombotic Syndrome. JAMA. 2016;315:1048-9. doi:

Reason: Guideline

18. Lin KW, Gostin LO. A Public Health Framework for Screening Mammography: Evidence-Based vs Politically Mandated Care. JAMA. 2016;315:977-8. doi: 10.1001/jama.2016.0322.

viewpoint

19. Kullo IJ, Rooke TW. CLINICAL PRACTICE. Peripheral Artery Disease. N Engl J Med. 2016;374:861-71. doi: 10.1056/NEJMcp1507631.

Reason: Mixture of case-report + review + guideline

20. Qaseem A, Barry MJ, Kansagara D; Clinical Guidelines Committee of the American College of Physicians. Nonpharmacologic Versus Pharmacologic Treatment of Adult Patients With Major Depressive Disorder: A Clinical Practice Guideline From the American College of Physicians. Ann Intern Med. 2016;164:350-9. doi: 10.7326/M15-2570.

Guideline

21. US Preventive Services Task Force (USPSTF)., Siu AL, Bibbins-Domingo K, Grossman DC, Baumann LC, Davidson KW, Ebell M, García FA, Gillman M, Herzstein J, Kemper AR, Krist AH, Kurth AE, Owens DK, Phillips WR, Phipps MG, Pignone MP. Screening for Impaired Visual Acuity in Older Adults: US Preventive Services Task Force Recommendation Statement. JAMA. 2016;315:908-14. doi: 10.1001/jama.2016.0763.

Reason: Statement

22. Singer M, Deutschman CS, Seymour CW, Shankar-Hari M, Annane D, Bauer M, Bellomo R, Bernard GR, Chiche JD, Coopersmith CM, Hotchkiss RS, Levy MM, Marshall JC, Martin GS, Opal SM, Rubenfeld GD, van der Poll T, Vincent JL, Angus DC. The Third International Consensus Definitions for Sepsis and Septic Shock (Sepsis-3). JAMA. 2016;315:801-10. doi: 10.1001/jama.2016.0287.

Reason: Consensus guidelines

23. Shankar-Hari M, Phillips GS, Levy ML, Seymour CW, Liu VX, Deutschman CS, Angus DC, Rubenfeld GD, Singer M; Sepsis Definitions Task Force.. Developing a New Definition and Assessing New Clinical Criteria for Septic Shock: For the Third International Consensus Definitions for Sepsis and Septic Shock (Sepsis-3). JAMA. 2016;315:775-87. doi: 10.1001/jama.2016.0289. Review.

Reason: Consensus guidelines

24. Friedman EM. VIDEOS IN CLINICAL MEDICINE. Removal of Foreign Bodies from the Ear and Nose. N Engl J Med. 2016;374:e7. doi: 10.1056/NEJMvcm1207469.

Reason: Video midia

25. Siu AL; US Preventive Services Task Force (USPSTF)., Bibbins-Domingo K, Grossman DC, Baumann LC, Davidson KW, Ebell M, García FA, Gillman M, Herzstein J, Kemper AR, Krist AH, Kurth AE, Owens DK, Phillips WR, Phipps MG, Pignone MP. Screening for Autism Spectrum Disorder in Young Children: US Preventive Services Task Force Recommendation Statement. JAMA. 2016;315:691-6. doi: 10.1001/jama.2016.0018.

Reason: Guideline

26. Schaeffer AJ, Nicolle LE. CLINICAL PRACTICE. Urinary Tract Infections in Older Men. N Engl J Med. 2016;374:562-71. doi: 10.1056/NEJMcp1503950.

Reason: Case-report

27. Grams ME, Sang Y, Levey AS, Matsushita K, Ballew S, Chang AR, Chow EK, Kasiske BL, Kovesdy CP, Nadkarni GN, Shalev V, Segev DL, Coresh J, Lentine KL, Garg AX; Chronic Kidney Disease Prognosis Consortium. Kidney-Failure Risk Projection for the Living Kidney-Donor Candidate. N Engl J Med. 2016;374:411-21. doi: 10.1056/NEJMoa1510491.

Reason: No review article

28. Cohen D. Rivaroxaban: can we trust the evidence? BMJ. 2016;352:i575. doi: 10.1136/bmj.i575.

Reason: Opinion article

29. Alkema L, Chou D, Hogan D, Zhang S, Moller AB, Gemmill A, Fat

DM, Boerma T, Temmerman M, Mathers C, Say L; United Nations

Maternal Mortality Estimation Inter-Agency Group collaborators and technical advisory group. Global, regional, and national levels and trends in maternal mortality between 1990 and 2015, with scenario-based projections to 2030: a systematic analysis by the UN Maternal Mortality Estimation Inter-Agency Group.Lancet. 2016;387:462-74. doi: 10.1016/S0140-6736(15)00838-7.

Reason: Other study design

30. Siu AL; US Preventive Services Task Force (USPSTF)., Bibbins-Domingo K, Grossman DC, Baumann LC, Davidson KW, Ebell M, García FA, Gillman M, Herzstein J, Kemper AR, Krist AH, Kurth AE, Owens DK, Phillips WR, Phipps MG, Pignone MP. Screening for Depression in Adults: US Preventive Services Task Force Recommendation Statement. JAMA. 2016;315:380-7. doi: 10.1001/jama.2015.18392.

Reason: Guideline

31. Black DM, Rosen CJ. Clinical Practice. Postmenopausal Osteoporosis. N Engl J Med. 2016;374:254-62. doi: 10.1056/NEJMcp1513724.

Reason: Case-report

32. Centor RM. Acute Uncomplicated Diverticulitis: What to Do Until We Have Better Data. Ann Intern Med. 2016;164:120-1. doi: 10.7326/M15-2499.

Reason: Opinion article

33. Stern T, Davis AM. Evaluation and Treatment of Patients With Constipation. JAMA. 2016;315:192-3. doi: 10.1001/jama.2015.16995.

Reason: guideline

34. Meissner HC. Viral Bronchiolitis in Children. N Engl J Med. 2016;374:62-72. doi: 10.1056/NEJMra1413456.

Reason: Case-report

35. Edelman EJ, Fiellin DA. In the Clinic. Alcohol Use. Ann Intern Med. 2016;164:ITC1-16. doi: 10.7326/AITC201601050.

Reason: not clear study design

36. Choudhry NK, Denberg TD, Qaseem A; Clinical Guidelines Committee of American College of Physicians. Improving Adherence to Therapy and Clinical Outcomes While Containing Costs: Opportunities From the Greater Use of Generic Medications: Best Practice Advice From the Clinical Guidelines Committee of the American College of Physicians. Ann Intern Med. 2016;164:41-9. doi: 10.7326/M14-2427.

Reason: guidelines

37. Shonin E, Van Gordon W, Griffiths MD. Does mindfulness work? BMJ. 2015;351:h6919. doi: 10.1136/bmj.h6919.

Reason: editorial

38. Mayor S. Meta-analysis finds no evidence for efficacy of nalmefene in treating alcohol

dependence. BMJ. 2015 Dec 29;351:h6988. doi: 10.1136/bmj.h6988.

Reason: Commentary

39. Rao VL, Cifu AS, Yang LW; American Gastroenterological Association. Pharmacologic Management of Irritable Bowel Syndrome. JAMA. 2015;314(24):2684-5. doi: 10.1001/jama.2015.16943.

Reason: Guideline

40. Gornall J. NICE told to back off "national policy issues". BMJ. 2015;351:h6766. doi: 10.1136/bmj.h6766.

Reason: Other study design

41. Daunton A, Langman G, Goulding JM. A cutaneous presentation of a common condition. BMJ. 2015;351:h6711. doi: 10.1136/bmj.h6711.

Reason. Case report

42. Ruegger J, Hodgkinson S, Field-Smith A, Ahmedzai SH; guideline

committee. Care of adults in the last days of life: summary of NICE guidance.BMJ. 2015;351:h6631. doi: 10.1136/bmj.h6631.

Reason: guideline

43. Burman KD, Wartofsky L. CLINICAL PRACTICE. Thyroid Nodules. N Engl J Med. 2015;373(24):2347-56. doi: 10.1056/NEJMcp1415786.

Reason: Case-report

44. Neilson J, O'Neill F, Dawoud D, Crean P; Guideline Development Group. Intravenous fluids in children and young people: summary of NICE guidance. BMJ. 2015;351:h6388. doi: 10.1136/bmj.h6388.

Reason: guideline

45. Siu AL; U S Preventive Services Task Force. Screening for Abnormal Blood Glucose and Type 2 Diabetes Mellitus: U.S.Preventive Services Task Force Recommendation Statement. Ann Intern Med. 2015;163(11):861-8. doi: 10.7326/M15-2345.

Reason: guideline

46. [No authors listed]. Treatment strategies for coronary in-stent restenosis: systematic review and hierarchical Bayesian network meta-analysis of 24 randomised trials and 4880

patients. BMJ. 2015;351:h6364. doi: 10.1136/bmj.h6364.

Reason: Erratum document

47. Hawkes N. Sixty seconds on . . . ADHD. BMJ. 2015;351:h6294. doi: 10.1136/bmj.h6294.

Reason: other study design

48. Sarri G, Davies M, Gholitabar M, Norman JE; Guideline Development

Group. Preterm labour: summary of NICE guidance. BMJ. 2015;351:h6283.

Reason: guideline

49. Stein MB, Sareen J. CLINICAL PRACTICE. Generalized Anxiety Disorder. N Engl J Med. 2015;373:2059-68. doi: 10.1056/NEJMcp1502514.

Reason: Case-report + management

50. Halpern SD. Toward Evidence-Based End-of-Life Care. N Engl J Med. 2015;373:2001-3. doi: 10.1056/NEJMp1509664.

Reason: Perspective article

51. Niven DJ, Gaudet JE, Laupland KB, Mrklas KJ, Roberts DJ, Stelfox HT. Accuracy of peripheral thermometers for estimating temperature: a systematic review and meta-analysis. Ann Intern Med. 2015;163:768-77. doi: 10.7326/M15-1150.

Reason: review not addressing disease

52. Siu AL; U.S. Preventive Services Task Force. Screening for high blood pressure in adults: U.S. Preventive Services Task Force recommendation statement. Ann Intern Med. 2015;163:778-86. doi: 10.7326/M15-2223.

Reason: guideline

53. Ross AG. Richer countries should help poorer ones plan for the next pandemic. BMJ. 2015;351:h6156. doi: 10.1136/bmj.h6156.

Reason: Personal view article

54. Dyer O.202. Tasers. BMJ. 2015;351:h6070. doi: 10.1136/bmj.h6070.

Reason: other type of article

55. Sarri G, Davies M, Lumsden MA; Guideline Development Group. Diagnosis and management of menopause: summary of NICE guidance. BMJ. 2015;351:h5746. doi: 10.1136/bmj.h5746.

Reason: guideline

56. Bangalore S, Maron DJ, Hochman JS. Evidence-Based Management of Stable Ischemic Heart Disease: Challenges and Confusion. JAMA. 2015;314(18):1917-8. doi: 10.1001/jama.2015.11219.

Reason: other ty of article (viewpoint)

57. Raja AS, Greenberg JO, Qaseem A, Denberg TD, Fitterman N, Schuur JD; Clinical Guidelines Committee of the American College of Physicians. Evaluation of Patients With Suspected Acute Pulmonary Embolism: Best Practice Advice From the Clinical Guidelines Committee of the American College of Physicians. Ann Intern Med. 2015;163:701-11. doi: 10.7326/M14-1772.

Reason: guideline

58. [No authors listed] Summaries for Patients. Evaluation of Patients With Suspected Acute Pulmonary Embolism: Best Practice Advice From the Clinical Guidelines Committee of the

American College of Physicians. Ann Intern Med. 2015 Nov 3;163(9):I34. doi: 10.7326/P15-9034.

Reason: guideline summary for patients

59. Schattman GL. CLINICAL PRACTICE. Cryopreservation of Oocytes. N Engl J Med. 2015;373(18):1755-60. doi: 10.1056/NEJMcp1307341.

Reason: combination of case-report + review + guideline

60. Fleetcroft R, Ford J, Gollop ND, MacKeith P, Perera K, Shafi. Difficulty accessing data from randomised trials of drugs for heart failure: a call for action. BMJ. 2015;351:h5002. doi: 10.1136/bmj.h5002.

Reason: methodological paper

61. Siu AL; U.S. Preventive Services Task Force. Behavioral and Pharmacotherapy Interventions for Tobacco Smoking Cessation in Adults, Including Pregnant Women: U.S. Preventive Services Task Force Recommendation Statement. Ann Intern Med. 2015;163:622-34. doi: 10.7326/M15-2023.

Reason: guideline

62. Patnode CD, Henderson JT, Thompson JH, Senger CA, Fortmann SP, Whitlock EP. Behavioral Counseling and Pharmacotherapy Interventions for Tobacco Cessation in adults, Including Pregnant Women: A Review of Reviews for the U.S. Preventive Services Task Force. Ann Intern Med. 2015;163:608-21. doi: 10.7326/M15-0171.

Reason: guideline

63. Carter M. Backlash against "pinkwashing" of breast cancer awareness campaigns. BMJ. 2015;351:h5399. doi: 10.1136/bmj.h5399.

Reason: commentary

64. Naci H, Lehman R, Wouters OJ, Goldacre B, Yudkin JS. Rethinking the appraisal and approval of drugs for type 2 diabetes. BMJ. 2015;351:h5260. doi: 10.1136/bmj.h5260.

Reason: analysis paper

65. Winkelman JW. CLINICAL PRACTICE. Insomnia Disorder. N Engl J Med. 2015;373:1437-44. doi: 10.1056/NEJMcp1412740.

Reason: combination of case report+review+guiodeline

66. Pollock K. Is home always the best and preferred place of death? BMJ. 2015;351:h4855. doi: 10.1136/bmj.h4855.

Reason: analysis paper

67. Niederman MS. In the Clinic: Community-Acquired Pneumonia. Ann Intern Med. 2015;163:ITC1-17. doi: 10.7326/AITC201510060.

In the Clinic: Community-Acquired Pneumonia.

Niederman MS.

PMID: 26436631 [PubMed - indexed for MEDLINE]

335. N Engl J Med. 2015 Jul 23;373(4):357-65. doi: 10.1056/NEJMra1411372.

Interventional Approaches to Gallbladder Disease.

Baron TH, Grimm IS, Swanstrom LL.

PMID: 26200981 [PubMed - indexed for MEDLINE]

67. Burns RB, Potter JE, Ricciotti HA, Reynolds EE. Screening Pelvic Examinations in Adult Women: Grand Rounds Discussion From the Beth Israel Deaconess Medical Center. Ann Intern Med. 2015;163:537-47. doi: 10.7326/M15-1220.

Reason: Analysis/discussion paper

68. Siu AL; U.S. Preventive Services Task Force. Screening for Iron Deficiency Anemia and Iron Supplementation in Pregnant Women to Improve Maternal Health and Birth Outcomes: U.S. Preventive Services Task Force Recommendation Statement. Ann Intern Med. 2015;163:529-36. doi: 10.7326/M15-1707.

Reason: guideline

69. Buchholz L. Exploring the Promise of Mindfulness as Medicine. JAMA. 2015;314:1327-9. doi: 10.1001/jama.2015.7023.

Reason: Perspective paper

70. Emdin CA, Anderson SG, Callender T, Conrad N, Salimi-Khorshidi G,

Mohseni H, Woodward M, Rahimi K. Usual blood pressure, peripheral arterial disease, and vascular risk: cohort study of 4.2 million adults. BMJ. 2015;351:h4865. doi: 10.1136/bmj.h4865.

Reason: other type of study design

71. Teicholz N. The scientific report guiding the US dietary guidelines: is it scientific? BMJ. 2015;351:h4962. doi: 10.1136/bmj.h4962.

Reason: analysis paper

72. McCarthy M. US panel narrows recommendations for low dose aspirin for cardiovascular disease prevention. BMJ. 2015;351:h4991. doi: 10.1136/bmj.h4991.

Reason: guideline

73. Wise J. T'ai chi benefits some patients with chronic conditions, review concludes. BMJ. 2015;351:h4968. doi: 10.1136/bmj.h4968.

Reason: other type of article

74. Davenport RJ. Review: Immediate vs deferred antiepileptics reduce recurrence at 1 to 2 y after an unprovoked first seizure. Ann Intern Med. 2015;163(6):JC8. doi: 10.7326/ACPJC-2015-163-6-008.

Reason: other type of article (summary of a review)

75. Chopra V, Flanders SA, Saint S, Woller SC, O'Grady NP, Safdar N, Trerotola SO,Saran R, Moureau N, Wiseman S, Pittiruti M, Akl EA, Lee AY, Courey A, Swaminathan L, LeDonne J, Becker C, Krein SL, Bernstein SJ; Michigan Appropriateness Guide for Intravenouse Catheters (MAGIC) Panel. The Michigan Appropriateness Guide for Intravenous Catheters (MAGIC): Results From a Multispecialty Panel Using the RAND/UCLA Appropriateness Method. Ann Intern Med. 2015;163(6 Suppl):S1-40. doi: 10.7326/M15-0744.

Reason: other type of article

76. Pronk NP, Remington PL; Community Preventive Services Task Force. Combined Diet and Physical Activity Promotion Programs for Prevention of Diabetes: Community Preventive Services Task Force Recommendation Statement. Ann Intern Med. 2015;163:465-8. doi: 10.7326/M15-1029.

Reason: guideline

77. Li R, Qu S, Zhang P, Chattopadhyay S, Gregg EW, Albright A, Hopkins D, Pronk NP. Economic Evaluation of Combined Diet and Physical Activity Promotion Programs to

Prevent Type 2 Diabetes Among Persons at Increased Risk: A Systematic Review for

the Community Preventive Services Task Force. Ann Intern Med. 2015;163(6):452-60. doi: 10.7326/M15-0469.

Reason: data on economic evaluation

78. Allen K, Pearson-Stuttard J, Hooton W, Diggle P, Capewell S,

O'Flaherty M.Potential of trans fats policies to reduce socioeconomic inequalities in mortality from coronary heart disease in England: cost effectiveness modelling study. BMJ. 2015;351:h4583. doi: 10.1136/bmj.h4583.

Reason: other type of study design

79. Chou D, Daelmans B, Jolivet RR, Kinney M, Say L; Every Newborn

Action Plan (ENAP) and Ending Preventable Maternal Mortality (EPMM) working groups. Ending preventable maternal and newborn mortality and stillbirths. BMJ. 2015;351:h4255. doi: 10.1136/bmj.h4255.

Reason: other type of study design

80. Branca F, Piwoz E, Schultink W, Sullivan LM. Nutrition and health in women, children, and adolescent girls. BMJ. 2015;351:h4173. doi: 10.1136/bmj.h4173.

Reason: Article not focused on the management of a disease

81. Daelmans B, Black MM, Lombardi J, Lucas J, Richter L, Silver K, Britto P, Yoshikawa H, Perez-Escamilla R, MacMillan H, Dua T, Bouhouch RR, Bhutta Z, Darmstadt GL, Rao N; steering committee of a new scientific series on early child development. Effective interventions and strategies for improving early child development.BMJ. 2015;351:h4029. doi: 10.1136/bmj.h4029.

Reason: Article not focused on the management of a disease

82. Downey L, Houten R, Murch S, Longson D; Guideline Development Group. Recognition, assessment, and management of coeliac disease: summary of updated NICE guidance. BMJ. 2015;351:h4513. doi: 10.1136/bmj.h4513.

Reason: guideline

83. Bannuru RR, McAlindon TE, Sullivan MC, Wong JB, Kent DM, Schmid CH. ffectiveness and Implications of Alternative Placebo Treatments: A Systematic Review and Network Meta-analysis of Osteoarthritis Trials. Ann Intern Med. 2015;163:365-72. doi: 10.7326/M15-0623.

Reason: methodological systematic review

84. Wilkin T. CLINICAL PRACTICE. Primary Care for Men Who Have Sex with Men. N Engl J Med. 2015;373:854-62. doi: 10.1056/NEJMcp1401303.

Reason: Case report + review + guideline

85. Amiel SA, Pursey N, Higgins B, Dawoud D; Guideline Development Group. Diagnosis and management of type 1 diabetes in adults: summary of updated NICE guidance. BMJ. 2015;351:h4188. doi: 10.1136/bmj.h4188.

Reason: guideline

86. Crowley RA, Kirschner N; Health and Public Policy Committee of the American College of Physicians. The integration of care for mental health, substance abuse, and other behavioral health conditions into primary care: executive summary of an American College of

Physicians position paper. Ann Intern Med. 2015;163(4):298-9. doi: 10.7326/M15-0510.

Reason: position paper

87. Downs JR, O'Malley PG. Management of dyslipidemia for cardiovascular disease risk reduction: synopsis of the 2014 U.S. Department of Veterans Affairs and U.S. Department of Defense clinical practice guideline. Ann Intern Med. 2015;163(4):291-7. doi: 10.7326/M15-0840.

Reason: guideline

88. Higgins JP, Welton NJ. Network meta-analysis: a norm for comparative effectiveness? Lancet. 2015;386:628-30. doi: 10.1016/S0140-6736(15)61478-7.

Reason: methodology study

89. MacKay CR, Torguson R, Waksman R. Delayed consent: will there be a shift in approach for US primary percutaneous coronary intervention trials? Lancet. 2015;386:714-6. doi: 10.1016/S0140-6736(15)60077-0.

Reason: editorial

90. Sedgwick P. What is publication bias in a meta-analysis? BMJ. 2015;351:h4419. doi: 10.1136/bmj.h4419.

Reason: methodology paper

91. Greer IA. CLINICAL PRACTICE. Pregnancy Complicated by Venous Thrombosis. N Engl J Med. 2015;373:540-7. doi: 10.1056/NEJMcp1407434.

Reson: Case-report+review+guideline

92. Rekart ML. Caring for sex workers. BMJ. 2015;351:h4011. doi: 10.1136/bmj.h4011.

Reason:not directly related to therapy of disease

93. Barua S, Greenwald R, Grebely J, Dore GJ, Swan T, Taylor LE. Restrictions for Medicaid Reimbursement of Sofosbuvir for the Treatment of Hepatitis C Virus Infection in the United States. Ann Intern Med. 2015;163:215-23. doi: 10.7326/M15-0406.

Reason: Not directly related to therapy of disease; economic perspective

94. Sumner SA, Mercy JA, Dahlberg LL, Hillis SD, Klevens J, Houry

D. Violence in the United States: Status, Challenges, and Opportunities. JAMA. 2015;314:478-88. doi: 10.1001/jama.2015.8371.

Reason: not related to therapy of disease

95. Ohtsuru A, Tanigawa K, Kumagai A, Niwa O, Takamura N, Midorikawa

S, Nollet K, Yamashita S, Ohto H, Chhem RK, Clarke M. Nuclear disasters and health: lessons learned, challenges, and proposals.Lancet. 2015;386:489-97. doi: 10.1016/S0140-6736(15)60994-1.

Reason: not related to therapy of disease

96. Xian Y, Wu J, O'Brien EC, Fonarow GC, Olson DM, Schwamm LH,

Bhatt DL, Smith EE, Suter RE, Hannah D, Lindholm B, Maisch L,

Greiner MA, Lytle BL, Pencina MJ, Peterson ED, Hernandez AF. Real world effectiveness of warfarin among ischemic stroke patients with atrial

fibrillation: observational analysis from Patient-Centered Research into Outcomes Stroke Patients Prefer and Effectiveness Research (PROSPER) study. BMJ. 2015;351:h3786. doi: 10.1136/bmj.h3786.

Reason: other study design

97. Luangasanatip N, Hongsuwan M, Limmathurotsakul D, Lubell Y, Lee

AS, Harbarth S, Day NP, Graves N, Cooper BS. Comparative efficacy of interventions to promote hand hygiene in hospital: systematic review and network meta-analysis. BMJ. 2015;351:h3728. doi: 10.1136/bmj.h3728.

Reason: not related to therapy of disease

98. Berens AE, Nelson CA. The science of early adversity: is there a role for large institutions in the care of vulnerable children? Lancet. 2015;386(9991):388-98. doi: 10.1016/S0140-6736(14)61131-4. Epub

2015 Jan 29.

Reason: not related to therapy of disease

99. Anderson C. Changing the way pharmacy only drugs are supplied would be unwise. BMJ. 2015;351:h3951. doi: 10.1136/bmj.h3951.

Reason: not related to therapy of disease

100. Grey A(1), Bolland M(2). Web of industry, advocacy, and academia in the management of osteoporosis.BMJ. 2015;351:h3170. doi: 10.1136/bmj.h3170.

Reason. Commentary

101. Moss JD, Cifu AS; ACC/AHA Task Force on Practice Guidelines. Management of Anticoagulation in Patients With Atrial Fibrillation. JAMA. 2015;314:291-2. doi: 10.1001/jama.2015.3088.

Reason: guideline

102. Shafei R, Goalby R, Hutchinson J. A case of breathlessness. BMJ. 2015;351:h3515. doi: 10.1136/bmj.h3515.

Reason: Case-report

103. Greinacher A. CLINICAL PRACTICE. Heparin-Induced Thrombocytopenia. N Engl J Med. 2015;373:252-61. doi: 10.1056/NEJMcp1411910.

Reason: Case-report+guideline+review

104. Fisher P, Ernst E. Should doctors recommend homeopathy? BMJ. 2015;351:h3735. doi: 10.1136/bmj.h3735.

Reason: Opinion paper

105. Benetos A, Rossignol P, Cherubini A, Joly L, Grodzicki T, Rajkumar

C, Strandberg TE, Petrovic M. Polypharmacy in the Aging Patient: Management of Hypertension in Octogenarians. JAMA. 2015;314(2):170-80. doi: 10.1001/jama.2015.7517.

Reason: Case-report + review + guideline

106. Piot P, Abdool Karim SS, Hecht R, Legido-Quigley H, Buse K, Stover

J, Resch S, Ryckman T, Møgedal S, Dybul M, Goosby E), Watts

C, Kilonzo N, McManus J, Sidibé M; UNAIDS–Lancet Commission. Defeating AIDS--advancing global health. Lancet. 2015;386:171-218. doi: 10.1016/S0140-6736(15)60658-4.

Reason: other study design/analysis paper

107. Sawleshwarkar S, Dwyer DE. Antivirals for herpes simplex viruses. BMJ. 2015;351:h3350. doi: 10.1136/bmj.h3350.

Reason: Case-report+review

108. Weeks AD, Neilson JP. Rethinking our approach to postpartum haemorrhage and uterotonics. BMJ. 2015;351:h3251. doi: 10.1136/bmj.h3251.

Reason: other study design/analysis paper

109. Reefhuis J, Devine O, Friedman JM, Louik C, Honein MA; National Birth Defects Prevention Study. Specific SSRIs and birth defects: Bayesian analysis to interpret new data in the context of previous reports. BMJ. 2015;351:h3190. doi: 10.1136/bmj.h3190.

Reason:Bayesian analysis from a specific sample

110. Andersson N, Nava-Aguilera E, Arosteguí J, Morales-Perez A, Suazo-Laguna H, Legorreta-Soberanis J, Hernandez-Alvarez C, Fernandez-Salas I, Paredes-Solís S, Balmaseda A, Cortés-Guzmán AJ, Serrano de Los Santos R, Coloma J, Ledogar RJ, Harris E. Evidence based community mobilization for dengue prevention in Nicaragua and Mexico (Camino Verde, the Green Way): cluster randomized controlled trial. BMJ. 2015;351:h3267. doi: 10.1136/bmj.h3267.

Reason: other study design

111. Shaw A, Bradley MD, Elyan S, Kurian KM. Tumour biomarkers: diagnostic, prognostic, and predictive. BMJ. 2015;351:h3449. doi: 10.1136/bmj.h3449.

Reason: case report+review

1123. Hawkes N. Cochrane reviews evidence on surgery for stress incontinence after controversy in Scotland. BMJ. 2015;351:h3578. doi: 10.1136/bmj.h3578.

Reason: other study design (overview of reviews)

113. Miller MR, Levy ML. Chronic obstructive pulmonary disease: missed diagnosis versus misdiagnosis. BMJ. 2015;351:h3021. doi: 10.1136/bmj.h3021.

Reason: other study design (analysis paper)

114. Holleman F, Uijldert M, Donswijk LF, Gale EA. Productivity of authors in the field of diabetes: bibliographic analysis of trial publications. BMJ. 2015;351:h2638. doi: 10.1136/bmj.h2638.

Reason: other study design (methodology paper)

115. Mehta RL, Cerdá J, Burdmann EA, Tonelli M, García-García G, Jha

V, Susantitaphong P, Rocco M, Vanholder R, Sever MS, Cruz D, Jaber B, Lameire NH, Lombardi R, Lewington A, Feehally J, Finkelstein F, Levin N, Pannu N, Thomas B, Aronoff-Spencer E, Remuzzi G. International Society of Nephrology's 0by25 initiative for acute kidney injury (zero preventable deaths by 2025): a human rights case for nephrology. Lancet. 2015;385:2616-43. doi: 10.1016/S0140-6736(15)60126-X. Epub

2015 Mar 13.

Reason: other study design

116. Mitchell SL. CLINICAL PRACTICE. Advanced Dementia. N Engl J Med. 2015;372:2533-40. doi: 10.1056/NEJMcp1412652.

Reason: case-report+review+guideline

117. Hill KP. Medical Marijuana for Treatment of Chronic Pain and Other Medical and Psychiatric Problems: A Clinical Review. JAMA. 2015;313(24):2474-83. doi: 10.1001/jama.2015.6199.

Reason. case-report+guideline+review

118. [No authors listed]. A GRADE Working Group approach for rating the quality of treatment effect estimates from network meta-analysis. BMJ. 2015;350:h3326. doi:

Reason: other study design

119. Whitlock EL, Kim H, Auerbach AD. Harms associated with single unit perioperative transfusion: retrospective population based analysis. BMJ. 2015;350:h3037. doi: 10.1136/bmj.h3037.

Reason: other study design

120. Mega JL, Stitziel NO, Smith JG, Chasman DI, Caulfield MJ, Devlin JJ, Nordio F, Hyde CL, Cannon CP, Sacks FM, Poulter NR, Sever PS, Ridker PM, Braunwald E, Melander O, Kathiresan S, Sabatine MS. Genetic risk, coronary heart disease events, and the clinical benefit of statin therapy: an analysis of primary and secondary prevention trials. ancet. 2015;385:2264-71. doi: 10.1016/S0140-6736(14)61730-X.

Reason: other study design

121. Padhi S(1), Glen J(1), Pordes BA(1), Thomas ME(2); Guideline Development Group. Management of anaemia in chronic kidney disease: summary of updated NICE guidance. BMJ. 2015;350:h2258. doi: 10.1136/bmj.h2258.

Reason: guideline

122. Hutton B, Salanti G, Caldwell DM, Chaimani A, Schmid CH, Cameron C, Ioannidis JP,

Straus S, Thorlund K, Jansen JP, Mulrow C, Catalá-López F, Gøtzsche PC, Dickersin

K, Boutron I, Altman DG, Moher D. The PRISMA extension statement for reporting of systematic reviews incorporating network meta-analyses of health care interventions: checklist and explanations. Ann Intern Med. 2015;162:777-84. doi: 10.7326/M14-2385.

Reason: other study design (methodological paper)

123. Harris RP. Incidental findings in the pancreas (and elsewhere): putting our patients (and

ourselves) in a difficult situation. Ann Intern Med. 2015;162:787-9. doi: 10.7326/M15-0590.

Reason: other study design (opinion paper)

124. Romeo GR, Abrahamson MJ. The 2015 standards for diabetes care: maintaining a patient-centered approach. Ann Intern Med. 2015;162(11):785-6. doi: 10.7326/M15-0385.

Reason: other study design (opinion paper)
