# Supplementary material for: A survey of prevalence of narrative and systematic reviews in five major medical journals
Source: BMC Med Res Methodol. 2017 Dec 28;17:176. doi: 10.1186/s12874-017-0453-y (PMC5746017; doi:10.1186/s12874-017-0453-y)
Supplement: Supplementary file 2 — Included articles. List of articles included in this study. (DOCX 61 kb) [file 12874_2017_453_MOESM2_ESM.docx]

**Additional file 2**

**List of included articles**

1. Alexander JH, Smith PK. Coronary-Artery Bypass Grafting. N Engl J Med. 2016;374:1954-64. doi: 10.1056/NEJMra1406944.
2. Amick HR, Gartlehner G, Gaynes BN, Forneris C, Asher GN, Morgan LC, Coker-Schwimmer E, Boland E, Lux LJ, Gaylord S, Bann C, Pierl CB, Lohr KN. Comparative benefits and harms of second generation antidepressants and cognitive behavioral therapies in initial treatment of major depressive disorder: systematic review and meta-analysis. BMJ. 2015;351:h6019. doi: 10.1136/bmj.h6019.
3. Andò G, Capodanno D. Radial Versus Femoral Access in Invasively Managed Patients With Acute Coronary. Ann Intern Med. 2015;163:932-40. doi: 10.7326/M15-1277.
4. Årdal C, Outterson K, Hoffman SJ, Ghafur A, Sharland M, Ranganathan N, Smith R, Zorzet A, Cohn J, Pittet D, Daulaire N, Morel C, Rizvi Z, Balasegaram M, Dar OA, Heymann DL, Holmes AH, Moore LS, Laxminarayan R, Mendelson M, Røttingen JA. International cooperation to improve access to and sustain effectiveness of antimicrobials. Lancet. 2016;387:296-307. doi: 10.1016/S0140-6736(15)00470-5.
5. Aslam AM, Patel AN. Facial cutaneous squamous cell carcinoma. BMJ. 2016;352:i1513. doi: 10.1136/bmj.i1513.
6. Attard G, Parker C, Eeles RA, Schröder F, Tomlins SA, Tannock I, Drake CG, de Bono JS. Prostate cancer. Lancet. 2016;387:70-82. doi: 10.1016/S0140-6736(14)61947-4
7. Badhiwala JH, Nassiri F, Alhazzani W, Selim MH, Farrokhyar F, Spears J, Kulkarni AV, Singh S, Alqahtani A, Rochwerg B, Alshahrani M, Murty NK, Alhazzani A, Yarascavitch B, Reddy K, Zaidat OO, Almenawer SA. Endovascular Thrombectomy for Acute Ischemic Stroke: A Meta-analysis. JAMA. 2015;314:1832-43. doi: 10.1001/jama.2015.13767
8. Balk EM, Earley A, Raman G, Avendano EA, Pittas AG, Remington PL. Combined Diet and Physical Activity Promotion Programs to Prevent Type 2 Diabetes Among Persons at Increased Risk: A Systematic Review for the Community Preventive Services Task Force. Ann Intern Med. 2015;163:437-51. doi: 10.7326/M15-0452.
9. Bang J, Spina S, Miller BL. Frontotemporal dementia. Lancet. 2015;386:1672-82. doi: 10.1016/S0140-6736(15)00461-4.
10. Bangalore S, Fakheri R, Toklu B, Messerli FH. Diabetes mellitus as a compelling indication for use of renin angiotensin system blockers: systematic review and meta-analysis of randomized trials. BMJ. 2016;352:i438. doi: 10.1136/bmj.i438.
11. Baron TH, Grimm IS, Swanstrom LL. Interventional Approaches to Gallbladder Disease. N Engl J Med. 2015;373:357-65. doi: 10.1056/NEJMra1411372.
12. Beasley R, Semprini A, Mitchell EA. Risk factors for asthma: is prevention possible? Lancet. 2015;386:1075-85. doi: 10.1016/S0140-6736(15)00156-7.
13. Berl T. Vasopressin antagonists. N Engl J Med. 2015;372:2207-16. doi: 10.1056/NEJMra1403672.
14. Bern C. Chagas' Disease. N Engl J Med. 2015;373:456-66. doi: 10.1056/NEJMra1410150.
15. Bernal W, Jalan R, Quaglia A, Simpson K, Wendon J, Burroughs A. Acute-on-chronic liver failure. Lancet. 2015 Oct 17;386:1576-87. doi: 10.1016/S0140-6736(15)00309-8.
16. Bertisch S. In the Clinic. Restless Legs Syndrome. Ann Intern Med. 2015;163:ITC1-11. doi: 10.7326/AITC201511030.
17. Bhangu A, Søreide K, Di Saverio S, Assarsson JH, Drake FT. Acute appendicitis: modern understanding of pathogenesis, diagnosis, and management. Lancet. 2015;386:1278-87. doi: 10.1016/S0140-6736(15)00275-5.
18. Bhatnagar R, Maskell N. The modern diagnosis and management of pleural effusions. BMJ. 2015;351:h4520. doi: 10.1136/bmj.h4520.
19. Biousse V, Newman NJ. Ischemic Optic Neuropathies. N Engl J Med. 2015;372:2428-36. doi: 10.1056/NEJMra1413352.
20. Bisson JI, Cosgrove S, Lewis C, Robert NP. Post-traumatic stress disorder. BMJ. 2015;351:h6161. doi: 10.1136/bmj.h6161.
21. Black RE, Levin C, Walker N, Chou D, Liu L, Temmerman M; DCP3 RMNCH Authors Group. Reproductive, maternal, newborn, and child health: key messages from Disease Control Priorities 3rd Edition. Lancet. 2016;388:2811-2824. doi: 10.1016/S0140-6736(16)00738-8.
22. Blinderman CD, Billings JA. Comfort Care for Patients Dying in the Hospital.N Engl J Med. 2015;373:2549-61. doi: 10.1056/NEJMra1411746.
23. Boehncke WH, Schön MP. Psoriasis. Lancet. 2015;386:983-94. doi: 10.1016/S0140-6736(14)61909-7.
24. Bolland MJ, Leung W, Tai V, Bastin S, Gamble GD, Grey A, Reid IR. Calcium intake and risk of fracture: systematic review. BMJ. 2015;351:h4580. doi: 10.1136/bmj.h4580.
25. Bolton JM, Gunnell D, Turecki G. Suicide risk assessment and intervention in people with mental illness. BMJ. 2015;351:h4978. doi: 10.1136/bmj.h4978.
26. Bonow RO, Leon MB, Doshi D, Moat N. Management strategies and future challenges for aortic valve disease. Lancet. 2016;387:1312-23. doi: 10.1016/S0140-6736(16)00586-9.
27. Brasure M, Fuchs E, MacDonald R, Nelson VA, Koffel E, Olson CM, Khawaja IS, Diem S, Carlyle M, Wilt TJ, Ouellette J, Butler M, Kane RL. Psychological and Behavioral Interventions for Managing Insomnia Disorder: An Evidence Report for a Clinical Practice Guideline by the American College of Physicians. Ann Intern Med. 2016;165:113-24. doi: 10.7326/M15-1782.
28. Bratton DJ, Gaisl T, Wons AM, Kohler M. CPAP vs Mandibular Advancement Devices and Blood Pressure in Patients With Obstructive Sleep Apnea: A Systematic Review and Meta-analysis. JAMA. 2015;314:2280-93. doi: 10.1001/jama.2015.16303.
29. Bruehl S. Complex regional pain syndrome. BMJ. 2015;351:h2730. doi: 10.1136/bmj.h2730.
30. Brunström M, Carlberg B. Effect of antihypertensive treatment at different blood pressure levels in patients with diabetes mellitus: systematic review and meta-analyses. BMJ. 2016;352:i717. doi: 10.1136/bmj.i717.
31. Bryce A, Hay AD, Lane IF, Thornton HV, Wootton M, Costelloe C. Global prevalence of antibiotic resistance in paediatric urinary tract infections caused by Escherichia coli and association with routine use of antibiotics in primary care: systematic review and meta-analysis. BMJ. 2016;352:i939. doi: 10.1136/bmj.i939.
32. Burch HB, Cooper DS. Management of Graves Disease: A Review. JAMA. 2015;314:2544-54. doi: 10.1001/jama.2015.16535.
33. Burrell JR, Halliday GM, Kril JJ, Ittner LM, Götz J, Kiernan MC, Hodges JR. The frontotemporal dementia-motor neuron disease continuum. Lancet. 2016;388:919-31. doi: 10.1016/S0140-6736(16)00737-6.
34. Cabanillas ME, McFadden DG, Durante C. Thyroid cancer. Lancet. 2016;388:2783-2795. doi: 10.1016/S0140-6736(16)30172-6.
35. Cahill TJ, Prendergast BD. Infective endocarditis. Lancet. 2016;387:882-93. doi: 10.1016/S0140-6736(15)00067-7.
36. Callaghan BC, Price RS, Feldman EL. Distal Symmetric Polyneuropathy: A Review. JAMA. 2015;314:2172-81. doi: 10.1001/jama.2015.13611.
37. Capitanio U, Montorsi F. Renal cancer. Lancet. 2016;387:894-906. doi: 10.1016/S0140-6736(15)00046-X.
38. Carey EJ, Ali AH, Lindor KD. Primary biliary cirrhosis. Lancet. 2015;386:1565-75. doi: 10.1016/S0140-6736(15)00154-3.
39. Cassese S, Byrne RA, Ndrepepa G, Kufner S, Wiebe J, Repp J, Schunkert H, Fusaro M, Kimura T, Kastrati A. Everolimus-eluting bioresorbable vascular scaffolds versus everolimus-eluting metallic stents: a meta-analysis of randomised controlled trials. Lancet. 2016;387:537-44. doi: 10.1016/S0140-6736(15)00979-4.
40. Chan E, Fogler JM, Hammerness PG. Treatment of Attention-Deficit/Hyperactivity Disorder in Adolescents: A Systematic Review. JAMA. 2016;315:1997-2008. doi: 10.1001/jama.2016.5453.
41. Chisolm MS, Payne JL. Management of psychotropic drugs during pregnancy. BMJ. 2016;532:h5918. doi: 10.1136/bmj.h5918.
42. Chou R, Dana T, Bougatsos C, Grusing S, Blazina I. Screening for Impaired Visual Acuity in Older Adults: Updated Evidence Report and Systematic Review for the US Preventive Services Task Force. JAMA. 2016;315:915-33. doi: 10.1001/jama.2016.0783.
43. Chou R, Hashimoto R, Friedly J, Fu R, Bougatsos C, Dana T, Sullivan SD, Jarvik J. Epidural Corticosteroid Injections for Radiculopathy and Spinal Stenosis: A Systematic Review and Meta-analysis. Ann Intern Med. 2015;163:373-81. doi: 10.7326/M15-0934.
44. Chua ML, Wee JT, Hui EP, Chan AT. Nasopharyngeal carcinoma. Lancet. 2016;387:1012-24. doi: 10.1016/S0140-6736(15)00055-0.
45. Chubak J, Whitlock EP, Williams SB, Kamineni A, Burda BU, Buist DS, Anderson ML. Aspirin for the Prevention of Cancer Incidence and Mortality: Systematic Evidence Reviews for the U.S. Preventive Services Task Force. Ann Intern Med. 2016;164:814-25. doi: 10.7326/M15-2117.
46. Chung KF. Targeting the interleukin pathway in the treatment of asthma. Lancet. 2015;386:1086-96. doi: 10.1016/S0140-6736(15)00157-9.
47. Clair DG, Beach JM. Mesenteric Ischemia. N Engl J Med. 2016;374:959-68. doi: 10.1056/NEJMra1503884.
48. Collis E, Mather H. Nausea and vomiting in palliative care. BMJ. 2015;351:h6249. doi: 10.1136/bmj.h6249.
49. Collister D, Komenda P, Hiebert B, Gunasekara R, Xu Y, Eng F, Lerner B, Macdonald K, Rigatto C, Tangri N. The Effect of Erythropoietin-Stimulating Agents on Health-Related Quality of Life in Anemia of Chronic Kidney Disease: A Systematic Review and Meta-analysis. Ann Intern Med. 2016;164:472-8. doi: 10.7326/M15-1839.
50. Compton WM, Jones CM, Baldwin GT. Relationship between Nonmedical Prescription-Opioid Use and Heroin Use. N Engl J Med. 2016;374:154-63. doi: 10.1056/NEJMra1508490.
51. Connor JP, Haber PS, Hall WD. Alcohol use disorders. Lancet. 2016;387:988-98. doi: 10.1016/S0140-6736(15)00122-1.
52. Crossley KM, Callaghan MJ, van Linschoten R. Patellofemoral pain. BMJ. 2015;351:h3939. doi: 10.1136/bmj.h3939.
53. Csete J, Kamarulzaman A, Kazatchkine M, Altice F, Balicki M, Buxton J, Cepeda J, Comfort M, Goosby E, Goulão J, Hart C, Kerr T, Lajous AM, Lewis S, Martin N, Mejía D, Camacho A, Mathieson D, Obot I, Ogunrombi A, Sherman S, Stone J, Vallath N, Vickerman P, Zábranský T, Beyrer C. Public health and international drug policy. Lancet. 2016;387:1427-1480. doi: 10.1016/S0140-6736(16)00619-X.
54. Cunha BA, Burillo A, Bouza E. Legionnaires' disease. Lancet. 2016;387:376-85. doi: 10.1016/S0140-6736(15)60078-2.
55. da Costa BR, Reichenbach S, Keller N, Nartey L, Wandel S, Jüni P, Trelle S. Effectiveness of non-steroidal anti-inflammatory drugs for the treatment of pain in knee and hip osteoarthritis: a network meta-analysis. Lancet. 2017;390:e21-e33. doi: 10.1016/S0140-6736(17)31744-0. (article was retracted)
56. Dalal HM, Doherty P, Taylor RS. Cardiac rehabilitation. BMJ. 2015;351:h5000. doi: 10.1136/bmj.h5000.
57. Dalbeth N, Merriman TR, Stamp LK. Gout. Lancet. 2016;388:2039-2052. doi: 10.1016/S0140-6736(16)00346-9.
58. Daniels PR. Peri-procedural management of patients taking oral anticoagulants. BMJ. 2015;351:h2391. doi: 10.1136/bmj.h2391.
59. Dar OA, Hasan R, Schlundt J, Harbarth S, Caleo G, Dar FK, Littmann J, Rweyemamu M, Buckley EJ, Shahid M, Kock R, Li HL, Giha H, Khan M, So AD, Bindayna KM, Kessel A, Pedersen HB, Permanand G, Zumla A, Røttingen JA, Heymann DL. Exploring the evidence base for national and regional policy interventions to combat resistance. Lancet. 2016;387:285-95. doi: 10.1016/S0140-6736(15)00520-6.
60. Dawes AJ, Maggard-Gibbons M, Maher AR, Booth MJ, Miake-Lye I, Beroes JM, Shekelle PG. Mental Health Conditions Among Patients Seeking and Undergoing Bariatric Surgery: A Meta-analysis. JAMA. 2016;315:150-63. doi: 10.1001/jama.2015.18118.
61. de Bernis L, Kinney MV, Stones W, Ten Hoope-Bender P, Vivio D, Leisher SH, Bhutta ZA, Gülmezoglu M, Mathai M, Belizán JM), Franco L, McDougall L, Zeitlin J, Malata A, Dickson KE, Lawn JE; Lancet Ending Preventable Stillbirths Series study group; Lancet Ending Preventable Stillbirths Series Advisory Group. Stillbirths: ending preventable deaths by 2030. Lancet. 2016;387:703-16. doi: 10.1016/S0140-6736(15)00954-X.
62. De Leo S, Lee SY, Braverman LE. Hyperthyroidism. Lancet. 2016;388:906-918. doi: 10.1016/S0140-6736(16)00278-6.
63. Dedert EA, McDuffie JR, Stein R, McNiel JM, Kosinski AS, Freiermuth CE, Hemminger A, Williams JW Jr. Electronic Interventions for Alcohol Misuse and Alcohol Use Disorders: A Systematic Review. Ann Intern Med. 2015;163:205-14. doi: 10.7326/M15-0285.
64. Delaney M, Wendel S, Bercovitz RS, Cid J, Cohn C, Dunbar NM, Apelseth TO, Popovsky M, Stanworth SJ, Tinmouth A, Van De Watering L, Waters JH, Yazer M, Ziman A; Biomedical Excellence for Safer Transfusion (BEST) Collaborative. Transfusion reactions: prevention, diagnosis, and treatment. Lancet. 2016;388:2825-2836. doi: 10.1016/S0140-6736(15)01313-6.
65. Dheda K, Barry CE 3rd, Maartens G. Tuberculosis. Lancet. 2016;387:1211-26. doi: 10.1016/S0140-6736(15)00151-8.
66. Dietz WH, Baur LA, Hall K, Puhl RM, Taveras EM, Uauy R. Management of obesity: improvement of health-care training and systems for prevention and care. Lancet. 2015;385:2521-33. doi: 10.1016/S0140-6736(14)61748-7.
67. Döhner H, Weisdorf DJ, Bloomfield CD. Acute Myeloid Leukemia. N Engl J Med. 2015;373:1136-52. doi: 10.1056/NEJMra1406184.
68. Drawz P, Rahman M. Chronic kidney disease. Ann Intern Med. 2015;162:ITC1-16. doi: 10.7326/AITC201506020.
69. Drug and Therapeutics Bulletin. The management of dry eye. BMJ. 2016;353:i2333. doi: 10.1136/bmj.i2333.
70. Early Breast Cancer Trialists' Collaborative Group (EBCTCG), Coleman R, Powles T, Paterson A, Gnant M, Anderson S, Diel I, Gralow J, von Minckwitz G, Moebus V, Bergh J, Pritchard KI, Bliss J, Cameron D, Evans V, Pan H, Peto R, Bradley R, Gray R. Adjuvant bisphosphonate treatment in early breast cancer: meta-analyses of individual patient data from randomised trials. Lancet. 2015;386:1353-61. doi: 10.1016/S0140-6736(15)60908-4.
71. Early Breast Cancer Trialists' Collaborative Group (EBCTCG), Dowsett M, Forbes JF, Bradley R, Ingle J, Aihara T, Bliss J, Boccardo F, Coates A, Coombes RC, Cuzick J, Dubsky P, Gnant M, Kaufmann M, Kilburn L, Perrone F, Rea D, Thürlimann B, van de Velde C, Pan H, Peto R, Davies C, Gray R. Aromatase inhibitors versus tamoxifen in early breast cancer: patient-level meta-analysis of the randomised trials. Lancet. 2015;386:1341-52. doi: 10.1016/S0140-6736(15)61074-1.
72. Ebell MH, Call M, Shinholser J, Gardner J. Does This Patient Have Infectious Mononucleosis?: The Rational Clinical Examination Systematic Review. JAMA. 2016;315:1502-9. doi: 10.1001/jama.2016.2111.
73. Eisman S, Sinclair R. Pityriasis rosea. BMJ. 2015;351:h5233. doi: 10.1136/bmj.h5233.
74. Elborn JS. Cystic fibrosis. Lancet. 2016;388:2519-2531. doi: 10.1016/S0140-6736(16)00576-6.
75. Ellul MA, Gholkar SA, Cross TJ. Hepatic encephalopathy due to liver cirrhosis. BMJ. 2015;351:h4187. doi: 10.1136/bmj.h4187.
76. Eng J, Wilson RF, Subramaniam RM, Zhang A, Suarez-Cuervo C, Turban S, Choi MJ, Sherrod C, Hutfless S, Iyoha EE, Bass EB. Comparative Effect of Contrast Media Type on the Incidence of Contrast-Induced Nephropathy: A Systematic Review and Meta-analysis. Ann Intern Med. 2016;164:417-24. doi: 10.7326/M15-1402.
77. Ettehad D, Emdin CA, Kiran A, Anderson SG, Callender T, Emberson J, Chalmers J, Rodgers A, Rahimi K. Blood pressure lowering for prevention of cardiovascular disease and death: a systematic review and meta-analysis. Lancet. 2016;387:957-67. doi: 10.1016/S0140-6736(15)01225-8.
78. Ezzedine K, Eleftheriadou V, Whitton M, van Geel N. Vitiligo. Lancet. 2015;386:74-84. doi: 10.1016/S0140-6736(14)60763-7.
79. Fink HA, Hemmy LS, MacDonald R, Carlyle MH, Olson CM, Dysken MW, McCarten JR, Kane RL, Garcia SA, Rutks IR, Ouellette J, Wilt TJ. Intermediate- and Long-Term Cognitive Outcomes After Cardiovascular Procedures in Older Adults: A Systematic Review. Ann Intern Med. 2015;163:107-17. doi: 10.7326/M14-2793.
80. Flenady V, Wojcieszek AM, Middleton P, Ellwood D, Erwich JJ, Coory M, Khong TY, Silver RM, Smith GC, Boyle FM, Lawn JE, Blencowe H, Leisher SH, Gross MM, Horey D, Farrales L, Bloomfield F, McCowan L, Brown SJ, Joseph KS, Zeitlin J, Reinebrant HE, Ravaldi C, Vannacci A, Cassidy J, Cassidy P, Farquhar C, Wallace E, Siassakos D, Heazell AE, Storey C, Sadler L, Petersen S, Frøen JF, Goldenberg RL; Lancet Ending Preventable Stillbirths study group; Lancet Stillbirths In High-Income Countries Investigator Group. Stillbirths: recall to action in high-income countries. Lancet. 2016;387:691-702. doi: 10.1016/S0140-6736(15)01020-X.
81. Floege J, Amann K. Primary glomerulonephritides. Lancet. 2016;387:2036-48. doi: 10.1016/S0140-6736(16)00272-5.
82. Forlino A, Marini JC. Osteogenesis imperfecta. Lancet. 2016;387:1657-71. doi: 10.1016/S0140-6736(15)00728-X.
83. Forman-Hoffman V, McClure E, McKeeman J, Wood CT, Middleton JC, Skinner AC. Screening for Major Depressive Disorder in Children and Adolescents: A Systematic Review for the U.S. Preventive Services Task Force. Ann Intern Med. 2016;164:342-9. doi: 10.7326/M15-2259.
84. Friedman D, Devinsky O. Cannabinoids in the Treatment of Epilepsy. N Engl J Med. 2015 ;373:1048-58. doi: 10.1056/NEJMra1407304.
85. Furuta GT, Katzka DA. Eosinophilic Esophagitis. N Engl J Med. 2015;373:1640-8. doi: 10.1056/NEJMra1502863.
86. Gelband H, Sankaranarayanan R, Gauvreau CL, Horton S, Anderson BO, Bray F, Cleary J, Dare AJ, Denny L, Gospodarowicz MK, Gupta S, Howard SC, Jaffray DA, Knaul F, Levin C, Rabeneck L, Rajaraman P, Sullivan T, Trimble EL, Jha P; Disease Control Priorities-3 Cancer Author Group. Costs, affordability, and feasibility of an essential package of cancer control interventions in low-income and middle-income countries: key messages from Disease Control Priorities, 3rd edition. Lancet. 2016;387:2133-2144. doi: 10.1016/S0140-6736(15)00755-2.
87. Giacoppo D, Gargiulo G, Aruta P, Capranzano P, Tamburino C, Capodanno D. Treatment strategies for coronary in-stent restenosis: systematic review and hierarchical Bayesian network meta-analysis of 24 randomised trials and 4880 patients. BMJ. 2015;351:h5392. doi: 10.1136/bmj.h5392.
88. Gibson PG, Vertigan AE. Management of chronic refractory cough. BMJ. 2015;351:h5590. doi: 10.1136/bmj.h5590.
89. Glyn-Jones S, Palmer AJ, Agricola R, Price AJ, Vincent TL, Weinans H, Carr AJ. Lancet. 2015;386:376-87. doi: 10.1016/S0140-6736(14)60802-3.
90. Goligher EC, Ferguson ND, Brochard LJ. Clinical challenges in mechanical ventilation. Lancet. 2016;387:1856-66. doi: 10.1016/S0140-6736(16)30176-3.
91. Gotts JE, Matthay MA. Sepsis: pathophysiology and clinical management. BMJ. 2016;353:i1585. doi: 10.1136/bmj.i1585.
92. Grande I, Berk M, Birmaher B, Vieta E. Bipolar disorder. Lancet. 2016;387:1561-72. doi: 10.1016/S0140-6736(15)00241-X.
93. Guirguis-Blake JM, Senger CA, Webber EM, Mularski RA, Whitlock EP. Screening for Chronic Obstructive Pulmonary Disease: Evidence Report and Systematic Review for the US Preventive Services Task Force. JAMA. 2016;315:1378-93. doi: 10.1001/jama.2016.2654.
94. Halland M, Saito YA. Irritable bowel syndrome: new and emerging treatments. BMJ. 2015;350:h1622. doi: 10.1136/bmj.h1622.
95. Han JH, Sullivan N, Leas BF, Pegues DA, Kaczmarek JL, Umscheid CA. Cleaning Hospital Room Surfaces to Prevent Health Care-Associated Infections: A Technical Brief. Ann Intern Med. 2015;163:598-607. doi: 10.7326/M15-1192.
96. Harnik IG. In the Clinic. Gastroesophageal Reflux Disease. Ann Intern Med. 2015;163:ITC1. doi: 10.7326/AITC201507070.
97. Harris AM, Hicks LA, Qaseem A; High Value Care Task Force of the American College of Physicians and for the Centers for Disease Control and Prevention. Appropriate Antibiotic Use for Acute Respiratory Tract Infection in Adults: Advice for High-Value Care From the American College of Physicians and the Centers for Disease Control and Prevention. Ann Intern Med. 2016;164:425-34. doi: 10.7326/M15-1840.
98. Hartmann LC, Lindor NM. The Role of Risk-Reducing Surgery in Hereditary Breast and Ovarian Cancer. N Engl J Med. 2016;374:454-68. doi: 10.1056/NEJMra1503523.
99. Hawkes C, Smith TG, Jewell J, Wardle J, Hammond RA, Friel S, Thow AM, Kain J. Smart food policies for obesity prevention. Lancet. 2015;385:2410-21. doi: 10.1016/S0140-6736(14)61745-1.
100. Hazlewood GS, Barnabe C, Tomlinson G, Marshall D, Devoe D, Bombardier C. Methotrexate monotherapy and methotrexate combination therapy with traditional and biologic disease modifying antirheumatic drugs for rheumatoid arthritis: abridged Cochrane systematic review and network meta-analysis. BMJ. 2016;353:i1777. doi: 10.1136/bmj.i1777.
101. Heazell AE, Siassakos D, Blencowe H, Burden C, Bhutta ZA, Cacciatore J, Dang N, Das J, Flenady V, Gold KJ, Mensah OK, Millum J, Nuzum D, O'Donoghue K, Redshaw M, Rizvi A, Roberts T, Toyin Saraki HE, Storey C, Wojcieszek AM, Downe S; Lancet Ending Preventable Stillbirths Series study group; Lancet Ending Preventable Stillbirths investigator group. Stillbirths: economic and psychosocial consequences. Lancet. 2016;387:604-16. doi: 10.1016/S0140-6736(15)00836-3.
102. Henry DA. In The Clinic: Hyponatremia. Ann Intern Med. 2015;163:ITC1-19. doi: 10.7326/AITC201508040.
103. Higham CE, Johannsson G, Shalet SM. Hypopituitarism. Lancet. 2016;388:2403-2415. doi: 10.1016/S0140-6736(16)30053-8.
104. Hole J, Hirsch M, Ball E, Meads C. Music as an aid for postoperative recovery in adults: a systematic review and meta-analysis. Lancet. 2015;386:1659-71. doi: 10.1016/S0140-6736(15)60169-6.
105. Holmes AH, Moore LS, Sundsfjord A, Steinbakk M, Regmi S, Karkey A, Guerin PJ, Piddock LJ. Understanding the mechanisms and drivers of antimicrobial resistance.Lancet. 2016;387:176-87. doi: 10.1016/S0140-6736(15)00473-0.
106. Horsburgh CR Jr, Barry CE 3rd, Lange C. Treatment of Tuberculosis. N Engl J Med. 2015;373:2149-60. doi: 10.1056/NEJMra1413919.
107. Huang TT, Cawley JH, Ashe M, Costa SA, Frerichs LM, Zwicker L, Rivera JA, Levy D, Hammond RA, Lambert EV, Kumanyika SK. Mobilisation of public support for policy actions to prevent obesity. Lancet. 2015;385:2422-31. doi: 10.1016/S0140-6736(14)61743-8.
108. Hunger SP, Mullighan CG. Acute Lymphoblastic Leukemia in Children. N Engl J Med. 2015;373:1541-52. doi: 10.1056/NEJMra1400972.
109. Husted SE, Ohman EM. Pharmacological and emerging therapies in the treatment of chronic angina. Lancet. 2015;386:691-701. doi: 10.1016/S0140-6736(15)61283-1.
110. Imazio M, Gaita F, LeWinter M. Evaluation and Treatment of Pericarditis: A Systematic Review. JAMA. 2015;314:1498-506. doi: 10.1001/jama.2015.12763.
111. Jayson GC, Kerbel R, Ellis LM, Harris AL. Antiangiogenic therapy in oncology: current status and future directions. Lancet. 2016;388:518-29. doi: 10.1016/S0140-6736(15)01088-0.
112. Jefferis JM, Connor AJ, Clarke MP. Amblyopia. BMJ. 2015;351:h5811. doi:10.1136/bmj.h5811.
113. Jiang L, Krumholz HM, Li X, Li J, Hu S. Achieving best outcomes for patients with cardiovascular disease in China by enhancing the quality of medical care and establishing a learning health-care system. Lancet. 2015;386:1493-505. doi: 10.1016/S0140-6736(15)00343-8.
114. Kahle KT, Kulkarni AV, Limbrick DD Jr, Warf BC. Hydrocephalus in children. Lancet. 2016;387:788-99. doi: 10.1016/S0140-6736(15)60694-8.
115. Kalia LV, Lang AE. Parkinson's disease. Lancet. 2015;386:896-912. doi: 10.1016/S0140-6736(14)61393-3.
116. Kamisawa T, Wood LD, Itoi T, Takaori K. Pancreatic cancer. Lancet. 2016;388:73-85. doi: 10.1016/S0140-6736(16)00141-0.
117. Kapadia BH, Berg RA, Daley JA, Fritz J, Bhave A, Mont MA. Periprosthetic joint infection. Lancet. 2016;387:386-94. doi: 10.1016/S0140-6736(14)61798-0.
118. Kelley AS, Morrison RS. Palliative Care for the Seriously Ill. N Engl J Med. 2015;373:747-55. doi: 10.1056/NEJMra1404684.
119. Kleopa KA. In the Clinic. Carpal Tunnel Syndrome. Ann Intern Med. 2015;163:ITC1. doi: 10.7326/AITC201509010.
120. Kotecha D, Manzano L, Krum H, Rosano G, Holmes J, Altman DG, Collins PD, Packer M, Wikstrand J, Coats AJ, Cleland JG, Kirchhof P, von Lueder TG, Rigby AS, Andersson B, Lip GY, van Veldhuisen DJ, Shibata MC, Wedel H, Böhm M, Flather MD; Beta-Blockers in Heart Failure Collaborative Group. Effect of age and sex on efficacy and tolerability of β blockers in patients with heart failure with reduced ejection fraction: individual patient data meta-analysis. BMJ. 2016;353:i1855. doi: 10.1136/bmj.i1855.
121. Krauss BS, Calligaris L, Green SM, Barbi E. Current concepts in management of pain in children in the emergency department. Lancet. 2016;387:83-92. doi: 10.1016/S0140-6736(14)61686-X.
122. Kularatne SA. Dengue fever. BMJ. 2015;351:h4661. doi: 10.1136/bmj.h4661.
123. Kullberg BJ, Arendrup MC. Invasive Candidiasis. N Engl J Med. 2015;373:1445-56. doi: 10.1056/NEJMra1315399.
124. Lacroix A, Feelders RA, Stratakis CA, Nieman LK. Cushing's syndrome. Lancet. 2015;386:913-27. doi: 10.1016/S0140-6736(14)61375-1.
125. Lambert N, Strebel P, Orenstein W, Icenogle J, Poland GA. Rubella. Lancet. 2015;385:2297-307. doi: 10.1016/S0140-6736(14)60539-0.
126. Lankisch PG, Apte M, Banks PA. Acute pancreatitis. Lancet. 2015;386:85-96. doi: 10.1016/S0140-6736(14)60649-8.
127. Laux P, Tralau T, Tentschert J, Blume A, Dahouk SA, Bäumler W, Bernstein E, Bocca B, Alimonti A, Colebrook H, de Cuyper C, Dähne L, Hauri U, Howard PC, Janssen P, Katz L, Klitzman B, Kluger N, Krutak L, Platzek T, Scott-Lang V, Serup J, Teubner W, Schreiver I, Wilkniß E, Luch A. A medical-toxicological view of tattooing. Lancet. 2016;387:395-402. doi: 10.1016/S0140-6736(15)60215-X.
128. Lawn JE, Blencowe H, Waiswa P, Amouzou A, Mathers C, Hogan D, Flenady V, Frøen JF, Qureshi ZU, Calderwood C, Shiekh S, Jassir FB, You D, McClure EM, Mathai M, Cousens S; Lancet Ending Preventable Stillbirths Series study group; Lancet Stillbirth Epidemiology investigator group. Stillbirths: rates, risk factors, and acceleration towards 2030. Lancet. 2016;387:587-603. doi: 10.1016/S0140-6736(15)00837-5.
129. Laxminarayan R, Matsoso P, Pant S, Brower C, Røttingen JA, Klugman K, Davies S. Access to effective antimicrobials: a worldwide challenge. Lancet. 2016;387:168-75. doi: 10.1016/S0140-6736(15)00474-2.
130. Lebwohl B, Ludvigsson JF, Green PH. Celiac disease and non-celiac gluten sensitivity. BMJ. 2015;351:h4347. doi: 10.1136/bmj.h4347.
131. Lee JS, Giesler DL, Gellad WF, Fine MJ. Antibiotic Therapy for Adults Hospitalized With Community-Acquired Pneumonia: A Systematic Review. JAMA. 2016;315:593-602. doi: 10.1001/jama.2016.0115.
132. Levy SD, Alladina JW, Hibbert KA, Harris RS, Bajwa EK, Hess DR. Lancet. 2016;387:1867-78. doi: 10.1016/S0140-6736(16)30245-8.
133. Li BZ, Threapleton DE, Wang JY, Xu JM, Yuan JQ, Zhang C, Li P, Ye QL, Guo B, Mao C, Ye DQ. Comparative effectiveness and tolerance of treatments for Helicobacter pylori: systematic review and network meta-analysis. BMJ. 2015;351:h4052. doi: 10.1136/bmj.h4052.
134. Lipska KJ, Krumholz H, Soones T, Lee SJ. Polypharmacy in the Aging Patient: A Review of Glycemic Control in Older Adults With Type 2 Diabetes. JAMA. 2016;315:1034-45. doi: 10.1001/jama.2016.0299.
135. Lobstein T, Jackson-Leach R, Moodie ML, Hall KD, Gortmaker SL, Swinburn BA, James WP, Wang Y, McPherson K. Child and adolescent obesity: part of a bigger picture. Lancet. 2015;385:2510-20. doi: 10.1016/S0140-6736(14)61746-3.
136. Lopez A, Cacoub P, Macdougall IC, Peyrin-Biroulet L. Iron deficiency anaemia. Lancet. 2016;387:907-16. doi: 10.1016/S0140-6736(15)60865-0.
137. Lumsden MA, Hamoodi I, Gupta J, Hickey M. Fibroids: diagnosis and management. BMJ. 2015;351:h4887. doi: 10.1136/bmj.h4887.
138. Lurie J, Tomkins-Lane C. Management of lumbar spinal stenosis. BMJ. 2016;352:h6234. doi: 10.1136/bmj.h6234.
139. Luzuriaga K, Mofenson LM. Challenges in the Elimination of Pediatric HIV-1 Infection. N Engl J Med. 2016;374:761-70. doi: 10.1056/NEJMra1505256.
140. Majumder S, Chari ST. Chronic pancreatitis. Lancet. 2016;387:1957-66. doi: 10.1016/S0140-6736(16)00097-0.
141. Mammen AL. Statin-Associated Autoimmune Myopathy. N Engl J Med. 2016;374:664-9. doi: 10.1056/NEJMra1515161.
142. Maraka S, Kennel KA. Bisphosphonates for the prevention and treatment of osteoporosis. BMJ. 2015;351:h3783. doi: 10.1136/bmj.h3783.
143. Maret-Ouda J, Brusselaers N, Lagergren J. What is the most effective treatment for severe gastro-oesophageal reflux disease? BMJ. 2015;350:h3169. doi: 10.1136/bmj.h3169.
144. Maruthur NM, Tseng E, Hutfless S, Wilson LM, Suarez-Cuervo C, Berger Z, Chu Y, Iyoha E, Segal JB, Bolen S. Diabetes Medications as Monotherapy or Metformin-Based Combination Therapy for Type 2 Diabetes: A Systematic Review and Meta-analysis. Ann Intern Med. 2016;164:740-51. doi: 10.7326/M15-2650.
145. Mathai SC, Danoff SK. Management of interstitial lung disease associated with connective tissue disease. BMJ. 2016;352:h6819. doi: 10.1136/bmj.h6819.
146. McIlvennan CK, Allen LA. Palliative care in patients with heart failure. BMJ. 2016;353:i1010. doi: 10.1136/bmj.i1010.
147. McPherson S, Lucey MR, Moriarty KJ. Decompensated alcohol related liver disease: acute management. BMJ. 2016;352:i124. doi: 10.1136/bmj.i124.
148. Mega JL, Simon T. Pharmacology of antithrombotic drugs: an assessment of oral antiplatelet and anticoagulant treatments. Lancet. 2015;386:281-91. doi: 10.1016/S0140-6736(15)60243-4.
149. Mendelson M, Røttingen JA, Gopinathan U, Hamer DH, Wertheim H, Basnyat B, Butler C, Tomson G, Balasegaram M. Maximising access to achieve appropriate human antimicrobial use in low-income and middle-income countries. Lancet. 2016;387:188-98. doi: 10.1016/S0140-6736(15)00547-4.
150. Miligkos M, Bannuru RR, Alkofide H, Kher SR, Schmid CH, Balk EM. Leukotriene-receptor antagonists versus placebo in the treatment of asthma in adults and adolescents: a systematic review and meta-analysis. Ann Intern Med. 2015;163:756-67. doi: 10.7326/M15-1059.
151. Minisola S, Pepe J, Piemonte S, Cipriani C. The diagnosis and management of hypercalcaemia. BMJ. 2015;350:h2723. doi: 10.1136/bmj.h2723.
152. Misra S, Oliver NS. Diabetic ketoacidosis in adults. BMJ. 2015;351:h5660. doi:10.1136/bmj.h5660.
153. Mohan C, Assassi S. Biomarkers in rheumatic diseases: how can they facilitate diagnosis and assessment of disease activity? BMJ. 2015;351:h5079. doi: 10.1136/bmj.h5079.
154. Mol BW, Roberts CT, Thangaratinam S, Magee LA, de Groot CJ, Hofmeyr GJ. Pre-eclampsia. Lancet. 2016;387:999-1011. doi: 10.1016/S0140-6736(15)00070-7.
155. Molnar AO, Fergusson D, Tsampalieros AK, Bennett A, Fergusson N, Ramsay T, Knoll GA. Generic immunosuppression in solid organ transplantation: systematic review and meta-analysis. BMJ. 2015;350:h3163. doi: 10.1136/bmj.h3163.
156. Morgan MS, Pearle MS. Medical management of renal stones. BMJ. 2016;352:i52. doi: 10.1136/bmj.i52.
157. Morice P, Leary A, Creutzberg C, Abu-Rustum N, Darai E. Endometrial cancer. Lancet. 2016;387:1094-108. doi: 10.1016/S0140-6736(15)00130-0.
158. Moritz ML, Ayus JC. Maintenance Intravenous Fluids in Acutely Ill Patients. N Engl J Med. 2015;373:1350-60. doi: 10.1056/NEJMra1412877.
159. Muzerengi S, Clarke CE. Initial drug treatment in Parkinson's disease. BMJ. 2015;351:h4669. doi: 10.1136/bmj.h4669.
160. Nable JV, Tupe CL, Gehle BD, Brady WJ. In-Flight Medical Emergencies during Commercial Travel. N Engl J Med. 2015;373:939-45. doi: 10.1056/NEJMra1409213.
161. Nasr H, Scriven JM. Superficial thrombophlebitis (superficial venous thrombosis). BMJ. 2015;350:h2039. doi: 10.1136/bmj.h2039.
162. Nathan DM. Diabetes: Advances in Diagnosis and Treatment. JAMA. 2015;314:1052-62. doi: 10.1001/jama.2015.9536.
163. Navarese EP, Kolodziejczak M, Schulze V, Gurbel PA, Tantry U, Lin Y, Brockmeyer M, Kandzari DE, Kubica JM, D'Agostino RB Sr, Kubica J, Volpe M, Agewall S, Kereiakes DJ, Kelm M. Effects of Proprotein Convertase Subtilisin/Kexin Type 9 Antibodies in Adults With Hypercholesterolemia: A Systematic Review and Meta-analysis. Ann Intern Med. 2015;163:40-51. doi: 10.7326/M14-2957.
164. Navari RM), Aapro M. Antiemetic Prophylaxis for Chemotherapy-Induced Nausea and Vomiting. N Engl J Med. 2016;374:1356-67. doi: 10.1056/NEJMra1515442.
165. NCD Risk Factor Collaboration (NCD-RisC). Worldwide trends in diabetes since 1980: a pooled analysis of 751 population-based studies with 4.4 million participants. Lancet. 2016;387:1513-30. doi: 10.1016/S0140-6736(16)00618-8.
166. Nelson HD, Fu R, Cantor A, Pappas M, Daeges M, Humphrey L. Effectiveness of Breast Cancer Screening: Systematic Review and Meta-analysis to Update the 2009 U.S. Preventive Services Task Force Recommendation. Ann Intern Med. 2016;164:244-55. doi: 10.7326/M15-0969.
167. Nelson HD, Pappas M, Cantor A, Griffin J, Daeges M, Humphrey L. Harms of Breast Cancer Screening: Systematic Review to Update the 2009 U.S. Preventive Services Task Force Recommendation. Ann Intern Med. 2016;164:256-67. doi: 10.7326/M15-0970.
168. Newby G, Bennett A, Larson E, Cotter C, Shretta R, Phillips AA, Feachem RG. The path to eradication: a progress report on the malaria-eliminating countries. The path to eradication: a progress report on the malaria-eliminating countries. Lancet. 2016;387:1775-84. doi: 10.1016/S0140-6736(16)00230-0.
169. Niederman MS. In the Clinic: Community-Acquired Pneumonia. Ann Intern Med. 2015;163:ITC1-17. doi: 10.7326/AITC201510060.
170. Nishimura RA, Vahanian A, Eleid MF, Mack MJ. Mitral valve disease--current management and future challenges. Lancet. 2016;387:1324-34. doi: 10.1016/S0140-6736(16)00558-4.
171. O'Brien JT, Thomas A. Vascular dementia. Lancet. 2015;386:1698-706. doi: 10.1016/S0140-6736(15)00463-8.
172. O'Connor E, Rossom RC, Henninger M, Groom HC, Burda BU. Primary Care Screening for and Treatment of Depression in Pregnant and Postpartum Women: Evidence Report and Systematic Review for the US Preventive Services Task Force. JAMA. 2016;315:388-406. doi: 10.1001/jama.2015.18948.
173. Ortblad KF, Salomon JA, Bärnighausen T, Atun R. Stopping tuberculosis: a biosocial model for sustainable development. Lancet. 2015;386:2354-62. doi: 10.1016/S0140-6736(15)00324-4.
174. Owen MJ, Sawa A, Mortensen PB. Schizophrenia. Lancet. 2016;388:86-97. doi: 10.1016/S0140-6736(15)01121-6.
175. Palmer BF, Clegg DJ. Electrolyte and Acid-Base Disturbances in Patients with Diabetes Mellitus. N Engl J Med. 2015;373:548-59. doi: 10.1056/NEJMra1503102.
176. Palmerini T, Benedetto U, Bacchi-Reggiani L, Della Riva D, Biondi-Zoccai G, Feres F, Abizaid A, Hong MK, Kim BK, Jang Y, Kim HS, Park KW, Genereux P, Bhatt DL, Orlandi C, De Servi S, Petrou M, Rapezzi C, Stone GW. Mortality in patients treated with extended duration dual antiplatelet therapy after drug-eluting stent implantation: a pairwise and Bayesian network meta-analysis of randomised trials. Lancet. 2015;385:2371-82. doi: 10.1016/S0140-6736(15)60263-X.
177. Patel N, Ram D, Swiderska N, Mewasingh LD, Newton RW, Offringa M. BMJ. 2015;351:h4240. doi: 10.1136/bmj.h4240.
178. Patel V, Chisholm D, Parikh R, Charlson FJ, Degenhardt L, Dua T, Ferrari AJ, Hyman S, Laxminarayan R, Levin C, Lund C, Medina Mora ME, Petersen I, Scott J, Shidhaye R, Vijayakumar L, Thornicroft G, Whiteford H; DCP MNS Author Group. Addressing the burden of mental, neurological, and substance use disorders: key messages from Disease Control Priorities, 3rd edition. Lancet. 2016;387:1672-85. doi: 10.1016/S0140-6736(15)00390-6.
179. Patel V, Xiao S, Chen H, Hanna F, Jotheeswaran AT, Luo D, Parikh R, Sharma E, Usmani S, Yu Y, Druss BG, Saxena S. The magnitude of and health system responses to the mental health treatment gap in adults in India and China. Lancet. 2016;388:3074-3084. doi: 10.1016/S0140-6736(16)00160-4.
180. Paul S, Saxena A, Terrin N, Viveiros K, Balk EM, Wong JB. Hepatitis B Virus Reactivation and Prophylaxis During Solid Tumor Chemotherapy: A Systematic Review and Meta-analysis. Ann Intern Med. 2016;164:30-40. doi: 10.7326/M15-1121.

Perrin EM, Viswanathan M. Screening for Major Depressive Disorder in Children and Adolescents: A Systematic Review for the U.S. Preventive Services Task Force. Ann Intern Med. 2016;164:342-9. doi: 10.7326/M15-2259.

1. Peyvandi F, Garagiola I, Young G. The past and future of haemophilia: diagnosis, treatments, and its complications. Lancet. 2016;388:187-97. doi: 10.1016/S0140-6736(15)01123-X.
2. Phadke VK, Bednarczyk RA, Salmon DA, Omer SB. Association Between Vaccine Refusal and Vaccine-Preventable Diseases in the United States: A Review of Measles and Pertussis. JAMA. 2016;315:1149-58. doi: 10.1001/jama.2016.1353.
3. Piccolo R, Giustino G, Mehran R, Windecker S. Stable coronary artery disease: revascularisation and invasive strategies. Lancet. 2015;386:702-13. doi: 10.1016/S0140-6736(15)61220-X.
4. Pillay J, Armstrong MJ, Butalia S, Donovan LE, Sigal RJ, Chordiya P, Dhakal S, Vandermeer B, Hartling L, Nuspl M, Featherstone R, Dryden DM. Behavioral Programs for Type 1 Diabetes Mellitus: A Systematic Review and Meta-analysis. Ann Intern Med. 2015;163:836-47. doi: 10.7326/M15-1399.
5. Pillay J, Armstrong MJ, Butalia S, Donovan LE, Sigal RJ, Vandermeer B, Chordiya P, Dhakal S, Hartling L, Nuspl M, Featherstone R, Dryden DM. Behavioral Programs for Type 2 Diabetes Mellitus: A Systematic Review and Network Meta-analysis. Ann Intern Med. 2015;163:848-60. doi: 10.7326/M15-1400.
6. Plourde G, Pancholy SB, Nolan J, Jolly S, Rao SV, Amhed I, Bangalore S, Patel T, Dahm JB, Bertrand OF. Radiation exposure in relation to the arterial access site used for diagnostic coronary angiography and percutaneous coronary intervention: a systematic review and meta-analysis. Lancet. 2015;386:2192-203. doi: 10.1016/S0140-6736(15)00305-0.
7. Plunkett A, Tong J. Sepsis in children. BMJ. 2015;350:h3017. doi: 10.1136/bmj.h3017.
8. Postma DS, Rabe KF. The Asthma-COPD Overlap Syndrome. N Engl J Med. 2015;373:1241-9. doi: 10.1056/NEJMra1411863.
9. Poulter NR, Prabhakaran D, Caulfield M. Hypertension. Lancet. 2015;386:801-12. doi: 10.1016/S0140-6736(14)61468-9.
10. Powell LW, Seckington RC, Deugnier Y. Haemochromatosis. Lancet. 2016;388:706-16. doi: 10.1016/S0140-6736(15)01315-X.
11. Prina E, Ranzani OT, Torres A. Community-acquired pneumonia. Lancet. 2015;386:1097-108. doi: 10.1016/S0140-6736(15)60733-4.
12. Prystowsky EN, Padanilam BJ, Fogel RI. Treatment of Atrial Fibrillation. JAMA. 2015;314:278-88. doi: 10.1001/jama.2015.7505.
13. Qian MB, Utzinger J, Keiser J, Zhou XN. Clonorchiasis. Lancet. 2016;387:800-10. doi: 10.1016/S0140-6736(15)60313-0.
14. Quon BS, Rowe SM. New and emerging targeted therapies for cystic fibrosis. BMJ. 2016;352:i859. doi: 10.1136/bmj.i859.
15. Rajpert-De Meyts E, McGlynn KA, Okamoto K, Jewett MA, Bokemeyer C. Testicular germ cell tumours. Lancet. 2016;387:1762-74. doi: 10.1016/S0140-6736(15)00991-5.
16. Rangaka MX, Cavalcante SC, Marais BJ, Thim S, Martinson NA, Swaminathan S, Chaisson RE. Controlling the seedbeds of tuberculosis: diagnosis and treatment of tuberculosis infection. Lancet. 2015;386:2344-53. doi: 10.1016/S0140-6736(15)00323-2.
17. Remenyi B, ElGuindy A, Smith SC Jr, Yacoub M, Holmes DR Jr. Valvular aspects of rheumatic heart disease. Lancet. 2016;387:1335-46. doi: 10.1016/S0140-6736(16)00547-X.
18. Richards JS, Dowell SM, Quinones ME, Kerr GS. How to use biologic agents in patients with rheumatoid arthritis who have comorbid disease. BMJ. 2015;351:h3658. doi: 10.1136/bmj.h3658.
19. Riddell A, Kennedy I, Tong CY. Management of sharps injuries in the healthcare setting. BMJ. 2015;351:h3733. doi: 10.1136/bmj.h3733.
20. Rinella ME. Nonalcoholic fatty liver disease: a systematic review. JAMA. 2015;313:2263-73. doi: 10.1001/jama.2015.5370.
21. Roberto CA, Swinburn B, Hawkes C, Huang TT, Costa SA, Ashe M, Zwicker L, Cawley JH, Brownell KD. Patchy progress on obesity prevention: emerging examples, entrenched barriers, and new thinking. Lancet. 2015;385:2400-9. doi: 10.1016/S0140-6736(14)61744-X.
22. Robinson BM, Akizawa T, Jager KJ, Kerr PG, Saran R, Pisoni RL. Factors affecting outcomes in patients reaching end-stage kidney disease worldwide: differences in access to renal replacement therapy, modality use, and haemodialysis practices. Lancet. 2016;388:294-306. doi: 10.1016/S0140-6736(16)30448-2.
23. Rodés-Cabau J, Taramasso M, O'Gara PT. Diagnosis and treatment of tricuspid valve disease: current and future perspectives. Lancet. 2016;388:2431-2442. doi: 10.1016/S0140-6736(16)00740-6.
24. Rodrigues FB, Neves JB, Caldeira D, Ferro JM, Ferreira JJ, Costa J. Endovascular treatment versus medical care alone for ischaemic stroke: systematic review and meta-analysis. BMJ. 2016;353:i1754. doi: 10.1136/bmj.i1754.
25. Rossignol P, Massy ZA, Azizi M, Bakris G, Ritz E, Covic A, Goldsmith D, Heine GH, Jager KJ, Kanbay M, Mallamaci F, Ortiz A, Vanholder R, Wiecek A, Zoccali C, London GM, Stengel B, Fouque D; ERA-EDTA EURECA-m working group; Red de Investigación Renal (REDINREN) network; Cardiovascular and Renal Clinical Trialists (F-CRIN INI-CRCT) network. The double challenge of resistant hypertension and chronic kidney disease. Lancet. 2015;386:1588-98. doi: 10.1016/S0140-6736(15)00418-3.
26. Rudmik L, Soler ZM. Medical Therapies for Adult Chronic Sinusitis: A Systematic Review. JAMA. 2015;314:926-39. doi: 10.1001/jama.2015.7544.
27. Salluh JI, Wang H, Schneider EB, Nagaraja N, Yenokyan G, Damluji A, Serafim RB, Stevens RD. Outcome of delirium in critically ill patients: systematic review and meta-analysis. BMJ. 2015;350:h2538. doi: 10.1136/bmj.h2538.
28. Salvo F, Moore N, Arnaud M, Robinson P, Raschi E, De Ponti F, Bégaud B, Pariente A. Addition of dipeptidyl peptidase-4 inhibitors to sulphonylureas and risk of hypoglycaemia: systematic review and meta-analysis. BMJ. 2016;353:i2231. doi: 10.1136/bmj.i2231.
29. Sanchez E, Vannier E, Wormser GP, Hu LT. Diagnosis, Treatment, and Prevention of Lyme Disease, Human Granulocytic Anaplasmosis, and Babesiosis: A Review. JAMA. 2016;315:1767-77. doi: 10.1001/jama.2016.2884.
30. Scammell TE. Narcolepsy. N Engl J Med. 2015;373:2654-62. doi: 10.1056/NEJMra1500587.
31. Scheltens P, Blennow K, Breteler MM, de Strooper B, Frisoni GB, Salloway S, Van der Flier WM. Alzheimer's disease. Lancet. 2016;388:505-17. doi: 10.1016/S0140-6736(15)01124-1.
32. Selph S, Dana T, Blazina I, Bougatsos C, Patel H, Chou R. Screening for type 2 diabetes mellitus: a systematic review for the U.S. Preventive Services Task Force. Ann Intern Med. 2015;162:765-76. doi: 10.7326/M14-2221.
33. Seymour CW, Rosengart MR. Septic Shock: Advances in Diagnosis and Treatment. JAMA. 2015;314:708-17. doi: 10.1001/jama.2015.7885.
34. Sharma T, Guski LS, Freund N, Gøtzsche PC. Suicidality and aggression during antidepressant treatment: systematic review and meta-analyses based on clinical study reports. BMJ. 2016;352:i65. doi: 10.1136/bmj.i65.
35. Shimbo D, Abdalla M, Falzon L, Townsend RR, Muntner P. Role of Ambulatory and Home Blood Pressure Monitoring in Clinical Practice: A Narrative Review. Ann Intern Med. 2015;163:691-700. doi: 10.7326/M15-1270.
36. Siemieniuk RA, Meade MO, Alonso-Coello P, Briel M, Evaniew N, Prasad M, Alexander PE, Fei Y, Vandvik PO, Loeb M, Guyatt GH. Corticosteroid Therapy for Patients Hospitalized With Community-Acquired Pneumonia: A Systematic Review and Meta-analysis. Ann Intern Med. 2015;163:519-28. doi: 10.7326/M15-0715.
37. Singh JA, Cameron C, Noorbaloochi S, Cullis T, Tucker M, Christensen R, Ghogomu ET, Coyle D, Clifford T, Tugwell P, Wells GA. Risk of serious infection in biological treatment of patients with rheumatoid arthritis: a systematic review and meta-analysis. Lancet. 2015;386:258-65. doi: 10.1016/S0140-6736(14)61704-9.
38. Siontis GC, Stefanini GG, Mavridis D, Siontis KC, Alfonso F, Pérez-Vizcayno MJ, Byrne RA, Kastrati A, Meier B, Salanti G, Jüni P, Windecker S. Percutaneous coronary interventional strategies for treatment of in-stent restenosis: a network meta-analysis. Lancet. 2015;386:655-64. doi: 10.1016/S0140-6736(15)60657-2.
39. Smith ME, Haney E, McDonagh M, Pappas M, Daeges M, Wasson N, Fu R, Nelson HD. Treatment of Myalgic Encephalomyelitis/Chronic Fatigue Syndrome: A Systematic Review for a National Institutes of Health Pathways to Prevention Workshop. Ann Intern Med. 2015;162:841-50. doi: 10.7326/M15-0114.
40. Smolen JS, Aletaha D, McInnes IB. Rheumatoid arthritis. Lancet. 2016;388:2023-2038. doi: 10.1016/S0140-6736(16)30173-8.
41. Smoliga JM, Weiss P, Rundell KW. Exercise induced bronchoconstriction in adults: evidence based diagnosis and management. BMJ. 2016;352:h6951. doi: 10.1136/bmj.h6951.
42. Solebo AL, Cumberland PM, Rahi JS. Whole-population vision screening in children aged 4-5 years to detect amblyopia. Lancet. 2015;385:2308-19. doi: 10.1016/S0140-6736(14)60522-5.
43. Søreide K, Thorsen K, Harrison EM, Bingener J, Møller MH, Ohene-Yeboah M, Søreide JA. Perforated peptic ulcer. Lancet. 2015;386:1288-98. doi: 10.1016/S0140-6736(15)00276-7.
44. Spencer FA, Prasad M, Vandvik PO, Chetan D, Zhou Q, Guyatt G. Longer- Versus Shorter-Duration Dual-Antiplatelet Therapy After Drug-Eluting Stent Placement: A Systematic Review and Meta-analysis. Ann Intern Med. 2015;163:118-26. doi: 10.7326/M15-0083.
45. Stam-Slob MC, Lambalk CB, van de Ree MA. Contraceptive and hormonal treatment options for women with history of venous thromboembolism. BMJ. 2015;351:h4847. doi: 10.1136/bmj.h4847.
46. Stark A, Donahue TR, Reber HA, Hines OJ. Pancreatic Cyst Disease: A Review. JAMA. 2016;315:1882-93. doi: 10.1001/jama.2016.4690.
47. Steenkamp MM, Litz BT, Hoge CW, Marmar CR. Psychotherapy for Military-Related PTSD: A Review of Randomized Clinical Trials. JAMA. 2015;314:489-500. doi: 10.1001/jama.2015.8370.
48. Storebø OJ, Krogh HB, Ramstad E, Moreira-Maia CR, Holmskov M, Skoog M, Nilausen TD, Magnusson FL, Zwi M, Gillies D, Rosendal S, Groth C, Rasmussen KB, Gauci D, Kirubakaran R, Forsbøl B, Simonsen E, Gluud C. Methylphenidate for attention-deficit/hyperactivity disorder in children and adolescents: Cochrane systematic review with meta-analyses and trial sequential analyses of randomised clinical trials. BMJ. 2015;351:h5203. doi: 10.1136/bmj.h5203.
49. Strum WB. Colorectal Adenomas. N Engl J Med. 2016;374:1065-75. doi: 10.1056/NEJMra1513581.
50. Sundaram V, Kowdley K. Management of chronic hepatitis B infection. BMJ. 2015;351:h4263. doi: 10.1136/bmj.h4263.
51. Swanton C, Govindan R. Clinical Implications of Genomic Discoveries in Lung Cancer. N Engl J Med. 2016;374:1864-73. doi: 10.1056/NEJMra1504688.
52. Swinburn B, Kraak V, Rutter H, Vandevijvere S, Lobstein T, Sacks G, Gomes F, Marsh T, Magnusson R. Strengthening of accountability systems to create healthy food environments and reduce global obesity. Lancet. 2015;385:2534-45. doi: 10.1016/S0140-6736(14)61747-5.
53. Tai V, Leung W, Grey A, Reid IR, Bolland MJ. Calcium intake and bone mineral density: systematic review and meta-analysis. BMJ. 2015;351:h4183. doi: 10.1136/bmj.h4183.
54. Talley NJ, Ford AC. Functional Dyspepsia. N Engl J Med. 2015;373:1853-63. doi: 10.1056/NEJMra1501505.
55. Tangri N, Grams ME, Levey AS, Coresh J, Appel LJ, Astor BC, Chodick G, Collins AJ, Djurdjev O, Elley CR, Evans M, Garg AX, Hallan SI, Inker LA, Ito S, Jee SH, Kovesdy CP, Kronenberg F, Heerspink HJ, Marks A, Nadkarni GN, Navaneethan SD, Nelson RG, Titze S, Sarnak MJ, Stengel B, Woodward M, Iseki K; CKD Prognosis Consortium. Multinational Assessment of Accuracy of Equations for Predicting Risk of Kidney Failure: A Meta-analysis. JAMA. 2016;315:164-74. doi: 10.1001/jama.2015.18202.
56. Thapar A, Cooper M. Attention deficit hyperactivity disorder. Lancet. 2016;387:1240-50. doi: 10.1016/S0140-6736(15)00238-X.
57. Theoharides TC, Valent P, Akin C. Mast Cells, Mastocytosis, and Related Disorders. N Engl J Med. 2015;373:163-72. doi: 10.1056/NEJMra1409760.
58. Theron G, Jenkins HE, Cobelens F, Abubakar I, Khan AJ, Cohen T, Dowdy DW. Data for action: collection and use of local data to end tuberculosis. Lancet. 2015;386:2324-33. doi: 10.1016/S0140-6736(15)00321-9.
59. Thorlund JB, Juhl CB, Roos EM, Lohmander LS. Arthroscopic surgery for degenerative knee: systematic review and meta-analysis of benefits and harms. BMJ. 2015;350:h2747. doi: 10.1136/bmj.h2747.
60. Thornicroft G, Mehta N, Clement S, Evans-Lacko S, Doherty M, Rose D, Koschorke M, Shidhaye R, O'Reilly C, Henderson C. Evidence for effective interventions to reduce mental-health-related stigma and discrimination. Lancet. 2016;387:1123-32. doi: 10.1016/S0140-6736(15)00298-6.
61. Tidey JW, Miller ME. Smoking cessation and reduction in people with chronic mental illness. BMJ. 2015;351:h4065. doi: 10.1136/bmj.h4065.
62. Timmis A. Acute coronary syndromes. BMJ. 2015;351:h5153. doi: 10.1136/bmj.h5153.
63. Tomkins A, Duff J, Fitzgibbon A, Karam A, Mills EJ, Munnings K, Smith S, Seshadri SR, Steinberg A, Vitillo R, Yugi P. Controversies in faith and health care. Lancet. 2015;386:1776-85. doi: 10.1016/S0140-6736(15)60252-5.
64. Towbin JA, Lorts A, Jefferies JL. Left ventricular non-compaction cardiomyopathy. Lancet. 2015;386:813-25. doi: 10.1016/S0140-6736(14)61282-4.
65. Trauer JM, Qian MY, Doyle JS, Rajaratnam SM, Cunnington D. Cognitive Behavioral Therapy for Chronic Insomnia: A Systematic Review and Meta-analysis. Ann Intern Med. 2015;163:191-204. doi: 10.7326/M14-2841.
66. Turecki G, Brent DA. Suicide and suicidal behaviour. Lancet. 2016;387:1227-39. doi: 10.1016/S0140-6736(15)00234-2.
67. Uthman OA, Okwundu C, Gbenga K, Volmink J, Dowdy D, Zumla A, Nachega JB. Optimal Timing of Antiretroviral Therapy Initiation for HIV-Infected Adults With Newly Diagnosed Pulmonary Tuberculosis: A Systematic Review and Meta-analysis. Ann Intern Med. 2015;163:32-9. doi: 10.7326/M14-2979.
68. Van Cutsem E, Sagaert X, Topal B, Haustermans K, Prenen H. Gastric cancer. Lancet. 2016;388:2654-2664. doi: 10.1016/S0140-6736(16)30354-3
69. Venekamp RP, Prasad V, Hay AD. Are topical antibiotics an alternative to oral antibiotics for children with acute otitis media and ear discharge? BMJ. 2016;352:i308. doi:10.1136/bmj.i308.
70. Verheugt FW, Granger CB. Oral anticoagulants for stroke prevention in atrial fibrillation: current status, special situations, and unmet needs. Lancet. 2015;386:303-10. doi: 10.1016/S0140-6736(15)60245-8.
71. Viale L, Allotey J, Cheong-See F, Arroyo-Manzano D, Mccorry D, Bagary M, Mignini L, Khan KS, Zamora J, Thangaratinam S; EBM CONNECT Collaboration. Epilepsy in pregnancy and reproductive outcomes: a systematic review and meta-analysis. Lancet. 2015;386:1845-52. doi: 10.1016/S0140-6736(15)00045-8.
72. Vieira ER, Palmer RC, Chaves PH. Prevention of falls in older people living in the community. BMJ. 2016;353:i1419. doi: 10.1136/bmj.i1419.
73. Volkow ND, McLellan AT. Opioid Abuse in Chronic Pain--Misconceptions and Mitigation Strategies. N Engl J Med. 2016;374:1253-63. doi: 10.1056/NEJMra1507771.
74. von Keudell AG, Weaver MJ, Appleton PT, Bae DS, Dyer GS, Heng M, Jupiter JB, Vrahas MS. Diagnosis and treatment of acute extremity compartment syndrome. Lancet. 2015;386:1299-310. doi: 10.1016/S0140-6736(15)00277-9.
75. Wald A. Constipation: Advances in Diagnosis and Treatment. JAMA. 2016;315:185-91. doi: 10.1001/jama.2015.16994.
76. Walker Z, Possin KL, Boeve BF, Aarsland D. Lewy body dementias. Lancet. 2015;386:1683-97. doi: 10.1016/S0140-6736(15)00462-6.
77. Wallis CJ, Mahar AL, Choo R, Herschorn S, Kodama RT, Shah PS, Danjoux C, Narod SA, Nam RK. Second malignancies after radiotherapy for prostate cancer: systematic review and meta-analysis. BMJ. 2016;352:i851. doi: 10.1136/bmj.i851.
78. Wang B, Kesselheim AS. Characteristics of efficacy evidence supporting approval of supplemental indications for prescription drugs in United States, 2005-14: systematic review. BMJ. 2015;351:h4679. doi: 10.1136/bmj.h4679.
79. Wanner C, Amann K, Shoji T. The heart and vascular system in dialysis. Lancet. 2016;388:276-84. doi: 10.1016/S0140-6736(16)30508-6.
80. Warkentin TE. Ischemic Limb Gangrene with Pulses. N Engl J Med. 2015;373:642-55. doi: 10.1056/NEJMra1316259.
81. Webb RH, Grant C, Harnden A. Acute rheumatic fever. BMJ. 2015;351:h3443. doi: 10.1136/bmj.h3443.
82. Wechalekar AD, Gillmore JD, Hawkins PN. Systemic amyloidosis. Lancet. 2016;387:2641-54. doi: 10.1016/S0140-6736(15)01274-X.
83. Weidinger S, Novak N. Atopic dermatitis. Lancet. 2016;387:1109-22. doi: 10.1016/S0140-6736(15)00149-X.
84. Whiting PF, Wolff RF, Deshpande S, Di Nisio M, Duffy S, Hernandez AV, Keurentjes JC, Lang S, Misso K, Ryder S, Schmidlkofer S, Westwood M, Kleijnen J. Cannabinoids for Medical Use: A Systematic Review and Meta-analysis. JAMA. 2015;313:2456-73. doi: 10.1001/jama.2015.6358.
85. Whitlock EP, Burda BU, Williams SB, Guirguis-Blake JM, Evans CV. Bleeding Risks With Aspirin Use for Primary Prevention in Adults: A Systematic Review for the U.S. Preventive Services Task Force. Ann Intern Med. 2016;164:826-35. doi: 10.7326/M15-2112.
86. Wiles KS, Jarvis S, Nelson-Piercy C. Are we overtreating subclinical hypothyroidism in pregnancy? BMJ. 2015;351:h4726. doi: 10.1136/bmj.h4726.
87. Willison HJ, Jacobs BC, van Doorn PA. Guillain-Barré syndrome. Lancet. 2016;388:717-27. doi: 10.1016/S0140-6736(16)00339-1.
88. Wilson MH, Habig K, Wright C, Hughes A, Davies G, Imray CH. Pre-hospital emergency medicine. Lancet. 2015;386:2526-34. doi: 10.1016/S0140-6736(15)00985-X.
89. Wilt TJ, MacDonald R, Brasure M, Olson CM, Carlyle M, Fuchs E, Khawaja IS, Diem S, Koffel E, Ouellette J, Butler M, Kane RL. Pharmacologic Treatment of Insomnia Disorder: An Evidence Report for a Clinical Practice Guideline by the American College of Physicians. Ann Intern Med. 2016;165:103-12. doi: 10.7326/M15-1781.
90. Wiviott SD, Steg PG. Clinical evidence for oral antiplatelet therapy in acute coronary syndromes. Lancet. 2015;386:292-302. doi: 10.1016/S0140-6736(15)60213-6.
91. Xie X, Atkins E, Lv J, Bennett A, Neal B, Ninomiya T, Woodward M, MacMahon S, Turnbull F, Hillis GS, Chalmers J, Mant J, Salam A, Rahimi K, Perkovic V, Rodgers A. Effects of intensive blood pressure lowering on cardiovascular and renal outcomes: updated systematic review and meta-analysis. Lancet. 2016;387:435-43. doi: 10.1016/S0140-6736(15)00805-3.
92. Yuen CM, Amanullah F, Dharmadhikari A, Nardell EA, Seddon JA, Vasilyeva I, Zhao Y, Keshavjee S, Becerra MC. Turning off the tap: stopping tuberculosis transmission through active case-finding and prompt effective treatment. Lancet. 2015;386:2334-43. doi: 10.1016/S0140-6736(15)00322-0.
93. Zaccardi F, Htike ZZ, Webb DR, Khunti K, Davies MJ. Benefits and Harms of Once-Weekly Glucagon-like Peptide-1 Receptor Agonist Treatments: A Systematic Review and Network Meta-analysis. Ann Intern Med. 2016;164:102-13. doi: 10.7326/M15-1432.
94. Ziff OJ, Lane DA, Samra M, Griffith M, Kirchhof P, Lip GY, Steeds RP, Townend J, Kotecha D. Safety and efficacy of digoxin: systematic review and meta-analysis of observational and controlled trial data. BMJ. 2015;351:h4451. doi: 10.1136/bmj.h4451.
95. Zumla A, Hui DS, Perlman S. Middle East respiratory syndrome. Lancet. 2015;386:995-1007. doi: 10.1016/S0140-6736(15)60454-8.
