# Supplementary material for: A survey of prevalence of narrative and systematic reviews in five major medical journals
Source: BMC Med Res Methodol. 2017 Dec 28;17:176. doi: 10.1186/s12874-017-0453-y (PMC5746017; doi:10.1186/s12874-017-0453-y)
Supplement: Supplementary file 3 — Systematic review definitions. Definitions of systematic reviews published in the literature. (DOCX 14 kb) [file 12874_2017_453_MOESM3_ESM.docx]

**Additional file 3**

**Systematic review definitions**

Haddaway et al. 2017

i) systematic reviews' methods should be described in sufficient detail to allow full repeatability and traceability; ii) they must include a systematic approach to identifying and screening relevant academic and grey literature, iii) they should include critical appraisal

of the validity (internal, i.e. quality, and external, i.e. generalisability) of included studies to give greater weight to more reliable studies (Higgins & Green, 2011; Steering Group of the Campbell Collaboration, 2015; The Collaboration for Environmental Evidence, 2013).

Page et al. 2016

A systematic review attempts to collate all relevant evidences that fits pre-specified eligibility criteria to answer a specific research question. It uses explicit, systematic methods to minimize bias in the identification, selection, synthesis, and summary of studies. When done well, this provides reliable findings from which conclusions can be drawn and decisions made [25, 26]. The key characteristics of a systematic review are (a) a clearly stated set of objectives with an explicit, reproducible methodology; (b) a systematic search that attempts to identify all studies that would meet the eligibility criteria; (c) an assessment of the validity of the findings of the included studies (e.g., assessment of risk of bias and confidence in cumulative estimates); and (d) systematic presentation, and synthesis, of the characteristics and findings of the included studies (PRISMA-P terminology)

Cochrane Collaboration (http://community.cochrane.org/glossary)

Systematic review (synonym: systematic overview)

A review of a clearly formulated question that uses systematic and explicit methods to identify, select, and critically appraise relevant research, and to collect and analyse data from the studies that are included in the review. Statistical methods (meta-analysis) may or may not be used to analyse and summarise the results of the included studies.
